# Socioeconomic inequalities in non-communicable disease risk factors in Botswana: a cross-sectional study

**DOI:** 10.1186/s12889-019-7405-x

**Published:** 2019-08-07

**Authors:** Mpho Keetile, Kannan Navaneetham, Gobopamang Letamo, Serai Daniel Rakgoasi

**Affiliations:** Department of Population Studies, University of Botswana, Gaborone, Botswana

**Keywords:** Inequalities, NCDs, Risk factors, Botswana, Decomposition analysis

## Abstract

**Background:**

The debate on socioeconomic inequalities in health dominates the research and policy agenda of many countries. The prevalence of non-communicable diseases (NCDs) is on the rise in recent years in Botswana. As a prevention and policy effort, the study provided an empirical evidence on socioeconomic inequalities in NCD risk factors in Botswana.

**Methods:**

Data used in this study was derived from a cross sectional survey on chronic non communicable diseases in Botswana conducted in 2016. The survey adopted a multistage sampling design and a sample of 1178 participants (males and females) aged 15 years and above was selected in both urban and rural areas of Botswana. The inequality analysis was conducted employing decomposition analysis using ADePT software version 6. Logistic regression models were used to show the association between NCD risk factors and socioeconomic status using SPSS version 25.

**Results:**

Concentration indices showed that poor physical activity (CI = 0.0546), alcohol consumption (CI = 0.1859) and overweight/obesity (CI = 0.038) were more concentrated among the non-poor while daily smoking (CI = − 0.0308) and poor fruit/vegetable consumption (CI = − 0.1909) were more concentrated among the poor. Wealth status was observed to be the leading contributor to socioeconomic inequality for daily smoking, poor fruit/vegetable consumption, overweight/obesity and poor physical activity. Education was the leading contributor to socioeconomic inequality for alcohol consumption.

**Conclusions:**

Findings in this study indicate the need for concerted differential efforts to address the needs of the poor and non-poor in order to reduce NCD risk factor inequalities.

**Electronic supplementary material:**

The online version of this article (10.1186/s12889-019-7405-x) contains supplementary material, which is available to authorized users.

## Background

It has been observed across the world that there are inequalities in health, no matter what measure of socioeconomic status has been used [1–3]. These inequalities have been found to be common among populations at both national and subnational levels. As a result understanding the impacts of social, economic and demographic factors on health is an important policy challenge. In some countries it has been observed that the burden of most non-communicable disease (NCD) risk factors is relatively higher amongst disadvantaged and marginalised individuals and groups, compared with those with higher socioeconomic status (SES) [1, 2]. As a consequence of this, there is well-documented evidence of inverse health and wealth gradient in many contexts [3, 4]. In some contexts the gradient between wealth and health has been found to be positive, while in other contexts it is negative [5].

Much of work on socioeconomic inequalities in NCD risk factors in high-income countries (HIC) has shown that the poor are more likely to report all NCD risk factors [6]. On the other hand studies on socioeconomic inequalities in low and middle-income countries (LMICs) regarding the interaction between SES and health are relatively scarce [7]. Few available studies from LMICs show that poor people are more likely to report smoking, poor fruit/vegetable consumption and alcohol consumption [8–12] and that overweight/obesity is more likely to be observed among the non-poor [13–17]. Available evidence also shows that there is no significant association between SES and poor physical activity [18].

Socioeconomic and behavioural factors have been recognized as some of the main explanatory variables for health inequalities [19, 20]. A significant part of socioeconomic differences in health have been linked to various health behaviours [21, 22]. The adoption of risky health behaviours have been observed to shift from people of higher to lower SES as countries develop more with higher socioeconomic groups adopting new behaviours early and discarding them rather quickly upon learning of the related health consequences [23]. As higher socioeconomic groups discard these risky behaviours, lower socioeconomic groups are more inclined to take up these behaviours later [23].

In Botswana NCD risk factor surveilaence has been ongoing in recent times. However, there is little evidence of the association between SES and the prevalence of NCD risk factors. It is important to undertsand socioeconomic differences in NCD risk factors in order to identify which SES groups are most affected. It has been observed that NCD risk factors have great variation in their distribution among different socioeconomic groups within societies, and their distribution patterns are quickly changing and they are taking diverse patterns within various societies [22–24]. Whereas there is some literature on social determinants of *NCDs*, little is known about the corresponding social determinants of *NCD risk factors*. Moreover, the majority of studies addressing the question of inequalities in NCD risk factors have often used conventional regression models [21–23] which are not the best-suited to estimate social inequalities in NCD risk factors or unveil their determinants. Consequently, more insight into inequalities in NCD risk factors is important for policy purposes and for providing an understanding of the factors that are likely to contribute to socioeconomic inequalities in Botswana. It is against this background, that this study aims to assess socioeconomic inequalities in NCD risk factors. We used decomposition analysis to determine the contribution of a broad range of social determinants on the NCD risk factors inequalities. This study would contribute to the enhanced understanding of the extent of NCD risk factors burden and their socioeconomic inequalities in Botswana.

## Methods

### Data

The study used secondary data derived from a cross sectional survey. The survey employed stratified multistage probability sampling technique to select respondents in three cities and towns, fifteen urban villages and fifteen rural areas across Botswana (refer to Additional file 1 methodology file). The survey was carried out in March 2016. The survey collected information on NCDs and their risk factors. The targeted sample size for the survey was 1280 from which 1178 respondents were successfully interviewed, yielding a response rate of 92%.

### Definitions and measurement of variables

Information on NCD risk factors was collected through self-reports. The survey questionnaire was adapted from the WHO STEPS and WHO Study on Global Ageing and Adult Health (SAGE) instruments (Additional file 2 questionnaire). Respondents were asked questions on tobacco use, alcohol consumption, physical activity and fruit and vegetable consumption. All NCD risk factors were dichotomised [24] to indicate whether respondents reported or did not report any NCD risk factors. For tobacco use respondents were asked whether they currently smoke tobacco products, yes=1 and no=0. Alcohol consumption was measured based on the intensity of alcohol consumed in the past 30 days. Respondents were asked (only those who reported to have consumed alcohol in the past 30 days) about the number of standard alcohol drinks they had each day in the past 7 days and if those who reported to have had three or more drinks per day (of approximately 60 g alcoho1) were considered to have exercisive drinking=1 and 0=otherwise.

An adapted version of the WHO STEPS questionnaire for adults was used to assess participants’ self-reported physical activity [25]. The questionnaire assessed physical activity done in the past 7 days in the domains of work and walking (includes at work and at home, walking to travel from place to place, and any other walking for recreation, sport, exercise, or leisure). The time spent on moderate and vigorous intensity activities was estimated in terms of frequency (days per week) and duration (minutes per day) taken in each of the two physical activity domains [25]. Poor physical activity variable was computed as daily minutes (min/day) of physical activity scores in the work and walking domains. The variable was computed by summing up the time spent (min/day) in moderate-intensity and vigorous-intensity activities across the two domains such that if respondents took >10 minutes bouts of physical activity per day and <10 minutess bouts of physical activity per day they were considered physical active=0 and to have poor physical activity=1, respectively.Poor fruit and vegetables consumption was created when an individual reported daily consumption of less than the recommended 5 servings of fruit and vegetables. Respondents reported the number of servings for fruits/vegetables they had in a typical day, and if the servings were less than 5 in a day, they were considered to be having poor fruit/vegetable consumption [26].

The NCD survey collected anthropometric information on height in meters (m) and weight in kilograms (kg) as per WHO guidelines [27]. Body Mass Index (BMI) was used to classify overweight/obesity. BMI was derived from weight and height: weight (kg) / (height (m) x height (m)) [1]. The Charder MS7301 250Kg digital scale and the Muac measuring tape were used for anthropometric measurements. Weight of respondents was measured, to the nearest 0.1 kg, while their height was measured in metres. BMI was categorized into: underweight (BMI < 18.5 kg/m^2^), normal weight (18.5 ≤ BMI < 25 kg/m^2^), overweight (25 ≤ BMI < 30 kg/m^2^) and obese (BMI ≥30 kg/m^2^) [1]. Overweight and obese were used to create a binary outcome variable which was coded as: being overweight/obese (BMI ≥ 25 kg/m^2^) =1; not overweight/obese =0 (BMI < 25 kg/m^2^).

A wealth index (WI) was constructed and used as a measure of socioeconomic status (SES). It is a composite measure, constructed from the indicators of ownership of consumer durables, housing characteristics, and access to public services. Information on a wide range of durable assets was collected (e. g. ownership of telephone, car, refrigerator, television), housing characteristics (e. g. material of dwelling floor and roof, main cooking fuel), access to basic services (e. g. electricity supply, source of drinking water, sanitation facilities) and ownership of livestock (e.g. cattle, goats, sheep, horses, chickens). Further to the collection of information on durable assets, information on land and livestock ownership was collected. Principal component analysis was employed to derive the wealth index variable, which had five categories from the 1st to the 5th quintile (poorest to richest).

#### Control variables

Socio-demographic factors such as age, sex, marital status, work status, residence and education were used as controls for the analysis. These variables were conceptualized to have an association with NCD risk factors on the basis of the review of literature. Therefore to control for their likely association with NCD risk factors, they were included in the combined effects model, so that the association between the wealth status and NCD risk factors becomes isolated and discernible.

### Statistical analysis

Logistic regression analysis was used to assess the association between socioeconomic variables and NCD risk factors using SPSS version 25. Results of logistic regression models were presented as adjusted odds ratios (AOR) together with their 95% confidence intervals.

Analysis of socioeconomic inequalities in NCD risk factors was done using ADePT software (version 6). The socioeconomic inequalities were derived using concentration curves and concentration indices. Concentration curves were used to plot the cumulative share of the NCD risk factor variables against the cumulative share of the wealth status variable. In calculating the cumulative percentages, wealth status was ranked from lowest to highest quintile. If any NCD risk factor was equally distributed, the curve would be running from the bottom left hand corner to the top right-hand corner (a 45° line). This is known as the line of equality. Contrarily, if the share of NCD risk factor was low among the poor, the concentration curve would lie below the line of equality [28–30]. The further the curve is from the diagonal, the greater the degree of inequality. The first case of socioeconomic inequality is the case in which individuals with high SES have a positive value of concentration index., while the second case, where the curve is above the diagonal line, is known as socioeconomic inequality which disadvantages the individuals of lower SES and the value of the concentration index is negative [31].

For the concentration index the value of the NCD risk factor assigned to each individual was taken to be a function of the socioeconomic category to which the individual belongs. The value of the concentration index ranges between − 1 to + 1. The index is 0 if there is no socioeconomic related inequality. The achievement index was also used with the concentration index to reflect the average level of NCD risk factors and the inequality in NCD risk factors between the poor and the better-off. It is the weighted average of the NCD risk factors of the various people in the sample, in which higher weights are attached to poorer people than to better-off people [29]. The larger value of the index is considered as higher health disachievment to one group of population than others group. (Refer to Additional file 3 for the decomposition analysis equation).

## Results

### Sample characteristics

Table 1 presents the characteristics of the study population. The study population constituted a high proportion of females (69.1%) than males (30.9%). The sample age distribution suggests a relatively young population, with over half (59%) of the sample being less than 39 years of age, and almost three quarters (73.5%) being less than fifty years of age. More than two fifths (45.4%) of the population resided in urban villages; just under a third (30.2%) resided in cities or towns while a quarter (24.5%) resided in rural settlements. Almost three quarters (73.8%) of respondents were never married; over a third (35.5%) had primary education or less; over a quarter (27.2%) had junior secondary education while just under a fifth had senior secondary education (17.3%) and tertiary education and over (19.9%). Close to two fifths (37.5%) of respondents were not employed; while over a quarter were employed in either the public (10.5%) or private sector (15.7%). Just over one in every ten (11.2%) were self-employed, while close to a fifth (18.8%) were either home makers or students; while under a tenth (6.4%) were retired from work.

**Table 1:** Socioeconomic characteristics of the study population (*N* = 1178) -NCD survey, 2016

Variable	Percentage (%)	Frequency (*N*)
Sex		
Male	30.9	364
Female	69.1	813
Missing		1
Age in years		
< 24	26.4	270
25–34	29.5	302
35–44	19.2	196
45–54	12.7	130
55–64	7.3	75
65+ years	4.9	50
Missing		155
Locality Type		
Cities/Towns	30.2	355
Urban Villages	45.4	534
Rural Settlements	24.5	288
Missing		1
Marital Status		
Never Married	73.8	864
Currently married	17	199
Formerly married	9.2	108
Missing		7
Highest Level of Education Attained		
Primary or Less	35.5	410
Junior Secondary	27.2	314
Senior Secondary	17.3	200
Tertiary & Over	19.9	230
Missing		24
Work Status in past 12 months		
Public Sector	10.5	122
Private Sector	15.7	182
Self Employed	11.2	130
Not Employed	37.5	436
Homemaker-Student	18.8	218
Retired-Other	6.4	74
Missing		16
Wealth status		
Lowest	19.9	234
Second	20.1	237
Middle	19.9	235
Fourth	20.1	237
Highest	19.9	235
Missing		***–***
Overall		1178

### Prevalence of NCD risk factors

**Fig. 1:**
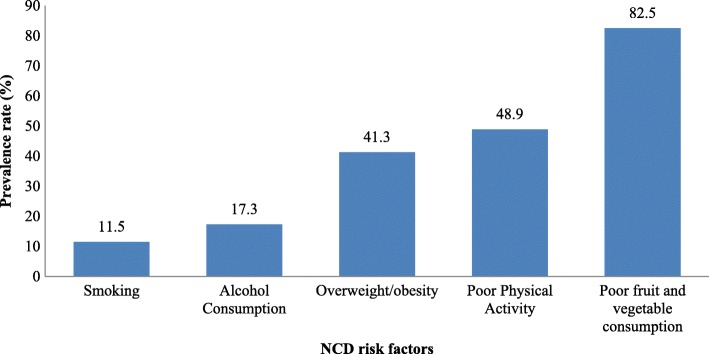
Prevalence of NCD risk factors in Botswana (*N* = 1178)-NCD survey, 2016

Figure 1 shows prevalence of NCD risk factors in the study population. Poor fruit and vegetable consumption was found to be the most prevalent NCD risk factor at 82.5%. Similarly poor physical activity (48.9%) and overweight/obesity (41.3%) were also found to be high. On the other hand it was seen that prevalence of smoking (11.5%) and alcohol consumption (17.3%) were relatively low in the population compared to other risk factors.

Table 2 presents the adjusted odds ratios (AORs) derived from logistic regression model for the risk factors among SES strata controlling for other demographic and social characteristics. The results indicate that the odds of smoking (AOR = 2.85, C.I. = 1.12–7.27), poor fruit and vegetable consumption (AOR = 2.30, C.I. = 1.06–5.86) and poor physical activity (AOR = 1.73, C.I. = 1.00–2.99) were significantly high among people with poor SES compared to highest SES. On the other hand,no significant differences observed between poor and non-poor for alcohol consumption and overweight/obesity.

**Table 2:** Odds ratios and 95% confidence intervals from logistic regressions of NCD risk factors among SES groups-NCD survey, 2016

SES/Wealth status	Smoking AOR CI	Alcohol consumption AOR CI	Poor fruit and vegetables AOR CI	Poor physical activity AOR CI	Overweight/obesity AOR CI
Lowest	2.85^a^ (1.12–7.27)	0.92 (0.30–2.82)	2.30^a^ (1.06–5.86)	1.73^a^ (1.00–2.99)	1.03 (0.64–1.68)
Second	1.37 (0.55–3.45)	1.59 (0.56–4.50	1.91^a^ (1.01–4.01)	1.62^a^ (1.00–2.67)	1.12 (0.68–1.85)
Middle	1.09 (0.45–2.66)	0.76 (0.30–2.82)	1.74^a^ (1.02–3.36)	1.38 (0.86–2.21)	1.20 (0.71–2.01)
Fourth	1.04 (0.44–2.47)	2.27^a^ (1.53–5.40)	1.43 (0.81–2.55)	1.44^a^ (1.02–2.20)	1.53 (0.87–2.69)
Highest	1.00	1.00	1.00	1.00	1.00

Note: *AOR* Adjusted Odd Ratios, *CI* Confidence Intervals, ^a^statistically significant at 5% level. Adjusted for sex, age, education, work status, marital status and residence. *N* = 1177

### Inequalities in NCDs risk factors

Results of the inequality measures of concentration indices (CI) and the standard achievement indices for NCD risk factors are presented in Table 3. When the concentration index is high, the achievement index will be low and vice versa [29]. The positive CI value of 0.1859 for alcohol consumption indicates skewness towards the non-poor population and the corresponding standard achievement index is low, while the low positive CI values of 0.0546 and 0.0308 shows that poor physical activity and overweight/obesity respectively, are close to the line of equality but seems to be concentrated among the non-poor. The negative CI values of − 0.0308 for daily smoking and − 0.1909 for poor fruit and vegetable consumption indicate skewness towards the poor population. These negative CI values (for poor fruit and vegetable consumption and smoking) are also accompanied by high corresponding standard achievement indices.

**Table 3:** Concentration indices showing inequalities in risk factors for NCDs in Botswana-NCD survey, 2016

NCD Risk Factors	Concentration Index (CI)	95% Confidence Interval	Standard Achievement Index
Poor physical activity	0.0546	(0.0251, 0.1231)	1.5069
Alcohol concumption	0.1859	(0.1103,0.4150)	0.1451
Daily smoking	−0.0308	(−0.1540, −0.0621)	0.0863
Poor fruit and vegetable consumption	−0.1909	(− 0.2112,- 0.023)	0.1126
Overweight/obesity	0.0308	(0.0123,0.3010)	1.303

**Fig. 2:**
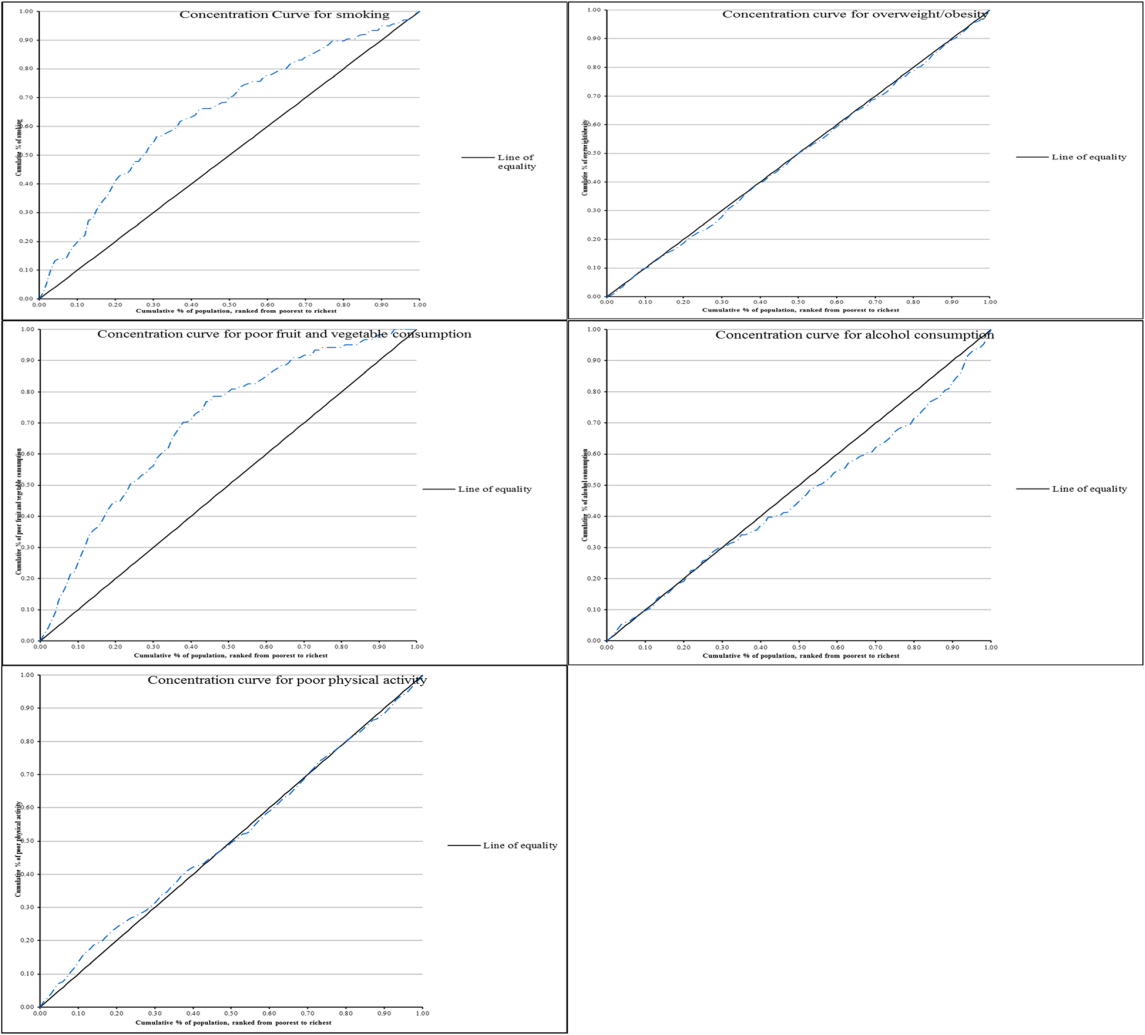
Concentration curves of risk factors for NCDs-NCD survey, 2016

Figure 2 shows the concentration curves plotting the cumulative share of selected NCD risk factors against the proportional cumulative share of wealth index (SES) score of individuals. The curve for alcohol consumption lies below the line of equality which confirms that alcohol consumption was more concentrated among the non-poor. Concentration curves for physical activity and overweight/obesity almost overlap with the line of equality showing that socioeconomic differences in physical activity and overweight/obesity were negligible but somewhat skewed towards the non-poor. This indicates that these two risk factors were skewed towards the non-poor although the inequality is relatively small. The curves for smoking and poor fruit and vegetable consumption were above the line of inequality showing concentration among the poor.

### Decomposing inequalities in risk factors for NCDs

**Fig. 3:**
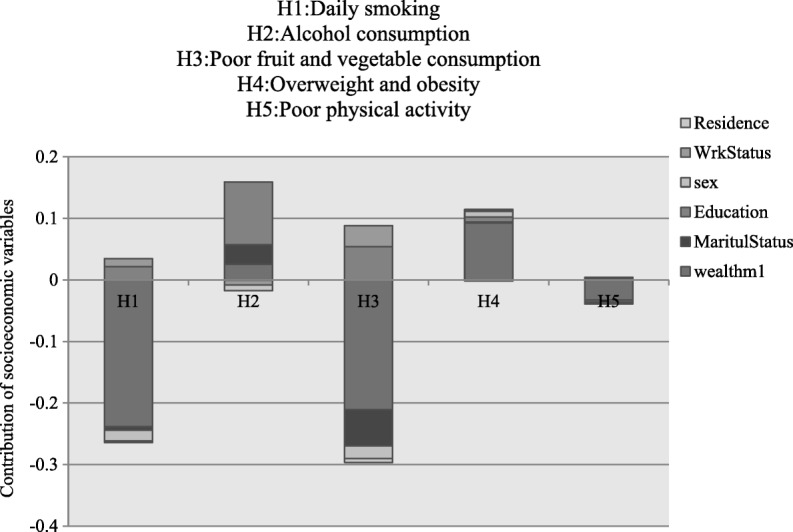
Decomposition of the concentration index for NCD risk factors in Botswana-NCD survey, 2016

Figure 3 presents a contribution of the selected socioeconomic determinants on the overall concentration index. The height of the bar corresponds to the contribution of socioeconomic factor (e.g. education, wealth status, sex, residence and work status) in explaining the observed inequality. Inequalities for daily smoking, poor physical activity, overweight/obesity and poor fruit and vegetable consumption were explained by differences in wealth status. The contribution of wealth status to daily smoking, poor physical activity and poor fruit and vegetable was negative while for overweight/obesity it was positive. Education was the leading contributor to socioeconomic differences for alcohol consumption and its contribution was positive indicating that alcohol consumption was high among the educated group.

## Discussion

Understanding inequalities in NCD risk factors from the Botswana perspective is particularly relevant since the social and public health landscape may blur any impressions of inqualities. For example the universal primary health care coverage and Botswana’s middle income status may suggest that there are no inequalities in health. Meanwhile, it has been observed in this study that alcohol consumption, overweight/obesity and poor physical activity were more concentrated among the non-poor while smoking and poor fruit and vegetable consumption were more concentrated among the poor. This corroborates results from other studies conducted in both developed and developing countries [31–33].

In the context of Botswana the concentration of alcohol among the non-poor is expected. This is because consumption of alcohol (especially commercial beverages) has been seen as a symbol of high SES [34]. Consequently, the poor often consume low-cost, homemade sorghum beer; while the non-poor usually take commercial beverages [33–35]. Evidence from study also corroborates some cross-sectional studies which have supported a positive relationship between SES and alcohol use, showing that individuals with higher SES engage in more frequent and heavier drinking of commercial beverages [36–38]. Although people with high SES have been found to consume greater amounts of alcohol compared with people with lower SES, the latter group seems to bear a disproportionate burden of negative alcohol-related consequences [36, 38].

The concentration curves for poor physical activity and overweight/obesity almost overlapped with the line of equality showing that although these risk factors were concentrated among the non-poor, the degree of inequality was small. The low inequality for poor physical activity and overweight/obesity may be explained by behavioural shift from traditionally active lifestyles to more industrialized and sedentary lifestyles among both the poor and non-poor in Botswana. Moreover, Botswana has experienced economic development and the consequent increases in income and the availability of inexpensive, high-calories foods, and low physical activity may have led to overweight/obesity which disproportionately affects the upper and middle classes to becoming widespread among the poor [35].

Concentration curves for daily smoking and poor fruit and vegetable consumption were above the line of equality suggesting that these two risk factors were more concentrated among the poor. This finding corroborates multivariate results which have also shown that smoking and poor fruit and vegetable consumption were more likely to be found among the poor. There is research evidence to suggest that purchasing and consumption of unhealthy diets, in particular, eating fewer fruits and vegetables, is strongly patterned by socioeconomic status (SES) [38–40]. It is suggested that poor fruit and vegetable consumption is more concentrated among the poor perhaps because the poor may not afford fruits and vegetables [40, 41]. Moreover, individuals of lower SES generally tend to have less healthy diets than those of higher SES [41].

As in previous studies it was found that smoking was disproportionately high among the poor [42–44]. It has been argued that due to high smoking rates among poor, they end up suffering more from diseases caused by smoking than do non-poor [43–46]. In Botswana the observed smoking inequality is rooted in many inequities^33^. This is because the poor have the least information about the health hazards of smoking, the fewest resources and social supports, and often the least access to services to help them quit. On top of that, the tobacco industry has a long history of targeting low SES individuals and communities.

Decomposition analysis showed that inequalities in alcohol consumption, poor physical activity and overweight/obesity can be explained by inequalities in education and wealth status. But the effects of contribution of education and wealth status to these three outcomes were different. This corroborates some studies which have pointed out education and wealth status as key determinants of health in developing countries [7, 16, 20, 23]. Similalry, inequalities in alcohol consumption were explained by inequalities in education, while for overweight/obesity and poor physical activity inequalities were explained by wealth status itself. However the contribution of wealth status for poor physical activity was negative implying the concentration among the poor.

### Strengths and limitations

The main limitation of this study is that NCD risk factors were based on self-reports. However since our study applies a standard chronic disease risk surveillance approach (WHO STEPs) the findings can be comparable to those from other setttings and countries. Since the NCD study sample was not designed to be representative of Botswana, a caution should be taken while generalizing these study findings.

## Conclusions

Overall the study showed mixed findings on the association between SES and NCD risk factors. It was noted that the poor were more likely to be exposed to some NCD risk factors than the non-poor and vice versa. For instance, smoking, poor physical activity, and poor fruit and vegetable were observed to be more concentrated among the poor, while alcohol consumption was found to be concentrated among the non-poor. These results demonstrate that socioeconomic factors play an important role in prevalence of NCD risk factors in Botswana. The recently introduced Botswana’s multisectoral national strategic plan implementation framework for combating NCDs (2017–2022) should focus on developing a socioeconomic status based awareness about the harmful impact of smoking, alcohol, physically inactivity and poor quality of diet facilited by comprehensive national monitoring and evaluvation framework. Considering high levels of smoking and poor fruit and vegetable consumption among the poor there is need for enhanced education on the long term effects of these risk factors. Similarly the non-poor need to be encouraged to adopt healthy diets and exercise regularly.

## Additional files

Additional file 1: Methodology for the NCDs study,2016 (Chapter 3) (DOCX 135 kb)

Additional file 2: Questionnaires (DOCX 68 kb)

Additional file 3: Decomposition analysis equation (DOCX 23 kb)

## Data Availability

Study materials and de-identified data that support the findings in this study may be obtained from Mpho Keetile on reasonable request through permission from the Office of Research and Development,University of Botswana.

## Abbreviations

AOR: Adjusted Odd Ratios
CI: Confidence Interval
NCD: Non-Communicable Diseases
SES: Socioeconomic Status
WHO: World Health Organization
