# Supplementary material for: Socioeconomic inequalities in non-communicable disease risk factors in Botswana: a cross-sectional study
Source: BMC Public Health. 2019 Aug 7;19:1060. doi: 10.1186/s12889-019-7405-x (PMC6686547; doi:10.1186/s12889-019-7405-x)
Supplement: Supplementary file 1 — Methodology for the NCDs study,2016 (Chapter 3) (DOCX 135 kb) [file 12889_2019_7405_MOESM1_ESM.docx]

# Additional file 1

# Appendix: Questionnaires

Department of Population Studies

University of Botswana

A Study on Non-Communicable Diseases in Botswana

Individual Questionnaire

March 2016

| **SURVEY INFORMATION** | |  | | | |  |
| --- | --- | --- | --- | --- | --- | --- |
| **1.0 Location and date** | | **Response** | | | | **Code** |
| 1001 | District Name (code) |  | | | |  |
| 1002 | Village Name (code) |  | | | |  |
| 1003 | Locality |  | | | |  |
| 1004 | Enumeration Area |  | | | |  |
| 1005 | Plot/house number |  | | | |  |
| 1006 | Interviewer ID |  | | | |  |
| 1007 | Consent has been read and obtained | Yes | | 1 | |  |
|  |  | No | | 2, If No, end of interview | |  |
|  |  | Interview Visits | | | |  |
|  |  | 1 | 2 | | 3 |  |
| 1008 | Date of the survey |  |  | |  |  |
| 1009 | Result* |  |  | |  |  |
| 1010 | Next Visit Date |  |  | |  |  |
| 1011 | Next Visit Time |  |  | |  |  |
| *Result Code | | Completed | | | 1 |  |
|  |  | Present but not available for interview | | | 2 |  |
|  |  | Postponed | | | 3 |  |
|  |  | Refused | | | 4 |  |
|  |  | Partially completed | | | 5 |  |
|  |  | Other (Specify) | | | 6 |  |
| **2.0 Interview Language and Name** | | **Response** | | | | **Code** |
| 1012 | Interview language | English | | | 1 |  |
|  |  | Setswana | | | 2 |  |
|  |  | Others | | | 3 |  |
| 1013 | Time of interview (24 hour clock) | _____ ________  Hours Minutes | | | |  |
| 1014 | Family Surname  Contact number (where possible) | First Name | | | |  |
| **Chronic Non-communicable Diseases in Botswana Study Questionnaire**  We are from the University of Botswana. We are working on a project concerned with Chronic non-communicable diseases (NCDs). Chronic non-communicable diseases (NCDs) have been observed to be on the increase across the world. These are long term medical conditions such as high blood pressure, diabetes, and others, which are not passed from one person to the other and have no known cure. They are controlled through taking long-term medication and making lifestyle changes. NCDs in Botswana have been increasing alarmingly over the last decade. Little is known on how many people in the country suffer from these diseases, how many people access and use the hospitals, clinics and other health services due to these diseases and the cost to the Government due to treating and managing these conditions in Botswana. The main objective of the study is to investigate patterns of chronic non-communicable diseases and their risk factors, health care utilization and health expenditure, and finally to examine how the life course factors influence the adult health. We would like to talk to you about this. The interview will take about 60 minutes. All the information we obtain will remain strictly confidential and your answers will never be identified. May I start now? | | | | | | |

| **SECTION 1: DEMOGRAPHIC INFORMATION** | | | | | |
| --- | --- | --- | --- | --- | --- |
| **Question and filters** | | **Coding categories** | | |  |
| 1015 | Sex (record male/female as observed) | Male | Female | |  |
| 1016 | What is your date of birth? | ___ ____ ______ [If known go to 1018]  dd mm year | | |  |
| 1017 | How old are you? | Years | _______ | |  |
| 1018 | In total how many years have you spent at school or in fulltime study (excluding preschool)? | Years | _______ | |  |
| 1019 | What is the highest level of education you have completed? (tick 1) | Non formal schooling | | 1 |  |
|  |  | Less than primary school | | 2 |  |
|  |  | Primary school completed | | 3 |  |
|  |  | Jnr. Secondary school completed | | 4 |  |
|  |  | Senior Secondary school completed | | 5 |  |
|  |  | High school completed | | 6 |  |
|  |  | Tertiary school completed | | 7 |  |
|  |  | College/University completed | | 8 |  |
|  |  | Postgraduate degree | | 9 |  |
|  |  | Refused | | 88 |  |
| 1020 | What is your (ethnic /racial group /cultural subgroup /others/Nationality) background? | Motswana | | 1 |  |
|  |  | Other African | | 2 |  |
|  |  | European | | 3 |  |
|  |  | Asian | | 4 |  |
|  |  | Others (Specify) | | 5 |  |
|  |  | Refused | | 88 |  |
| 1021 | What is your current marital status? | Never married | | 1 |  |
|  |  | Currently married | | 2 |  |
|  |  | Separated | | 3 |  |
|  |  | Divorced | | 4 |  |
|  |  | Widowed | | 5 |  |
|  |  | Not married but Living with partner/cohabit | | 6 |  |
|  |  | Refused | | 88 |  |

| 1022 | Which of the following best describes your main work status over the past 12 months? | Government employee | | 1 |  |
| --- | --- | --- | --- | --- | --- |
|  |  | Non-government employee | | 2 |  |
|  |  | Self-employed | | 3 |  |
|  |  | Non-paid/unpaid family helper | | 4 |  |
|  |  | Student | | 5 |  |
|  |  | Homemaker/house work | | 6 |  |
|  |  | Retired | | 7 |  |
|  |  | Unemployed (able to work) | | 8 |  |
|  |  | Unemployed (unable to work) | | 9 |  |
|  |  | Other (Specify) | | 87 |  |
|  |  | Refused | | 88 |  |
| 1023 | How many people including yourself, live in your household? | Less than 15  15-64  65 and above | |  |  |
| 1024 | Taking the past year, can you tell me what the total earnings of the household have been in Pula?  *RECORD ONLY ONE, NOT ALL 3* | Per week | ___ __ _ | |  |
|  |  | OR per month | _______ | |  |
|  |  | OR per year | _______ | |  |
|  |  | Refused |  | |  |
| 1025 | If you don’t know the amount, can you give an estimate of the annual household income if I read some options to you? Is it  *READ OPTIONS* | <5,000 Pula | | 1 |  |
|  |  | 5,000 – 9,000 | | 2 |  |
|  |  | 10,000 – 14,000 | | 3 |  |
|  |  | 15,000 – 19,000 | | 4 |  |
|  |  | ≥ 20,000 | | 5 |  |
|  |  | Don't Know | | 77 |  |
|  |  | Refused | | 88 |  |

| **SECTION 2: HOUSING CHARACTERISTICS**  I would like to ask you some questions about your dwelling or home. | | | | | | | |
| --- | --- | --- | --- | --- | --- | --- | --- |
| 2001 | Is this dwelling where you live…?  INTERVIEWER: *read options to the Respondent.* | 1. Owned by the household head and fully paid off 2. Owned by the household head but not yet fully paid for 3. Owned by someone else in household and fully paid off 4. Owned by someone else in household but not yet fully paid off 5. Rented………………………… 6. Provided free of charge…………. 7. Other (Specify): | | | | |  |
| 2002 | How many rooms does this dwelling have in total, without counting the bathrooms/toilets or hallways/passage ways? |  | | | | |  |
| 2003 | Does any member of this household own land used for planting? | Yes | | 1 | | |  |
|  |  | No | | 2 | | |  |
| 2004 | How was the land used for planting acquired? | Land-board | | 01 | | |  |
|  |  | Tribal | | 02 | | |  |
|  |  | Inheritance | | 03 | | |  |
|  |  | Freehold | | 04 | | |  |
|  |  | Lease | | 05 | | |  |
|  |  | TGLP | | 06 | | |  |
|  |  | Syndicate | | 07 | | |  |
|  |  | Employer/Relative | | 08 | | |  |
|  |  | Self-allocated | | 09 | | |  |
| **Assets and Household Income**  **PERMANENT INCOME INDICATORS (ASSETS)** | | | | | | | |
| I would like to ask you a few more questions about your home and items you might have in your home. Remember that any information you provide will be kept confidential. | | | | | | | |
| 2003a | How many televisions are there in your household?  *(If none enter “00”)* | | ___________________ | | | |  |
| 2004a | Do you have a security system in your home? | | 1 Yes | | | 2 No |  |
| 2005 | How many cars are there in your household?  *(If none enter “00”)* | | ___________________ | | | |  |
| 2006 | Does your home have electricity? | | 1 Yes | | 2 No | |  |
| 2007 | Does your household or anyone in your household have…? | | | | | |  |
|  | A Bicycle? | | 1 Yes | | 2 No | |  |
|  | A Van/bakkie? | | 1 Yes | | 2 No | |  |
|  | A motor car? | | 1 Yes | | 2 No | |  |
|  | A tractor? | | 1 Yes | | 2 No | |  |
|  | A wheel barrow? | | 1 Yes | | 2 No | |  |
|  | A built-in kitchen sink? | | 1 Yes | | 2 No | |  |
|  | Employ someone in house who is not a member of family | | 1 Yes | | 2 No | |  |
|  | A washing machine? | | 1 Yes | | 2 No | |  |
|  | A dishwasher? | | 1 Yes | | 2 No | |  |
|  | A refrigerator? | | 1 Yes | | 2 No | |  |
|  | Hot running water? | | 1 Yes | | 2 No | |  |
|  | A mobile/cellular telephone? | | 1 Yes | | 2 No | |  |
|  | A donkey cart /animal drawn cart or sled? | | 1 Yes | | 2 No | |  |
|  | A computer? (Desktop/laptop) | | 1 Yes | | 2 No | |  |
|  | A Hi-Fi or music centre (stereo system)? | | 1 Yes | | 2 No | |  |
|  | Internet access in the home? | | 1 Yes | | 2 No | |  |
|  | A motorbike? | | 1 Yes | | 2 No | |  |
|  | A television set? | | 1 Yes | | 2 No | |  |
|  | A radio? | | 1 Yes | | 2 No | |  |
|  | A second home? | | 1 Yes | | 2 No | |  |
| 2008 | How many cattle are there? If none 00 | |  | | | |  |
| 2009 | How many goats are there? If none 00 | |  | | | |  |
| 2010 | How many sheep are there? If none 00 | |  | | | |  |
| 2011 | How many pigs are there? If none 00 | |  | | | |  |
| 2012 | How many chickens (poultry) are there? If none 00 | |  | | | |  |
| 2013 | How many donkeys/mules are there? If none 00 | |  | | | |  |
| 2014 | How many horses are there? If none 00 | |  | | | |  |
| 2015 | How many ostrich are there? If none 00 | |  | | | |  |
| 2016 | How many game are there? If none 00 | |  | | | |  |
| 2017 | Would you say your household's financial situation is…? | | Very Bad  Bad  Moderate  Good  Very Good | | | |  |

| **SECTION 3: RISK FACTORS** | | | | | | | | |
| --- | --- | --- | --- | --- | --- | --- | --- | --- |
| **Tobacco use:** Now I am going to ask you some questions about tobacco use. | | | | | | | | |
| 3001 | 1. Do you currently smoke any tobacco products such as cigarettes, cigars, or pipes? 2. Do you use snuff, chewed tobacco? | | 1: daily | 2: yes but not daily | | | 3: No not at all, | ***If 2 or No, go to Q3004*** |
| 3002 | For how many years have you been smoking daily? | | _________ | | | | |  |
| 3003 | On average, how many of the following products do you smoke each day? | | Manufactured cigarettes | | | |  |  |
|  |  |  | Hand-rolled cigarette | | | |  |  |
|  |  |  | Pipe full of tobacco | | | |  |  |
|  |  |  | Others (e.g. cigars etc) | | | |  |  |
| **Alcohol use:** Now I am going to ask you some questions about Alcohol use. | | | | | | | | |
| 3004 | Have you ever consumed a drink that contains alcohol (such as beer, wine, spirits, chibuku, homemade brews, khadi, mokuru, fermented cider etc.)? | 1. Yes 2. Never | | | | | | ***If 2 or Never, go to Q3007*** |
| 3005a | Have you ever consumed alcohol in the past 30 days? | 1. Yes 2. No | | | | | |  |
| 3005b | During the past 12 months, **how frequently** have you had at least one alcoholic drink? | 1. Daily 2. 5-6 days per week 3. 3-4 days per week 4. 1-2 days per week 5. 1-3 days per month 6. Less than once a month | | | | | |  |
| 3006 | During the past 7 days, how many standard drinks of any alcoholic beverage did you have each day?  1 glass of beer  1 small glass of wine  1 shot of whisky | Monday | | | 1 | | |  |
|  |  | Tuesday | | | 2 | | |  |
|  |  | Wednesday | | | 3 | | |  |
|  |  | Thursday | | | 4 | | |  |
|  |  | Friday | | | 5 | | |  |
|  |  | Saturday | | | 6 | | |  |
|  |  | Sunday | | | 7 | | |  |
| **Nutrition**  The next questions ask about the fruits and vegetables that you usually eat. I have a nutrition card here that shows you some examples of local fruits and vegetables. Each picture represents the size of a serving. As you answer these questions please think of a typical week in the last year. | | | | | | | | |
| **Question** | | **Response** | | | | | |  |
| 3007 | Do you eat **fruits** at least one times in a **week**? | Yes  No | | | | 1  2 | | ***If No, go to Q3010*** |
| 3008 | In a typical week, on how many days do you **eat fruit**? | Number of days Don't Know 77 | | | | *└─┴─┘* | |  |
| 3009 | How many **servings** of fruit do you eat on **one** of those days? | Number of servings  Don't Know 77 | | | | └─┴─┘ | |  |

| 3010 | If no what is the reason for **not eating fruits**? | Not available | | 1 | |  |
| --- | --- | --- | --- | --- | --- | --- |
|  |  | Not affordable/expensive | | 2 | |  |
|  |  | Gives me discomfort | | 3 | |  |
|  |  | Seasonal | | 4 | |  |
|  |  | Others (Specify) | | 5 | |  |
|  |  | Don’t know/No reason | | 77 | |  |
| 3011 | Do you eat vegetables at least one times in a week? | Yes  No | | 1  2 | | ***If No, go to Q3014*** |
| 3012 | In a typical week, on how many days do you **eat vegetables**? | Number of days Don't Know 77 | | *└─┴─┘* | | ***If Zero days, go to Q3015*** |
| 3013 | How many **servings** of vegetables do you eat on one of those days? | Number of servings  Don’t know 77 | | └─┴─┘ | |  |
| 3014 | If no what is the reason for **not eating vegetables**? | Not available | | 1 | |  |
|  |  | Not affordable/expensive | | 2 | |  |
|  |  | Gives me discomfort | | 3 | |  |
|  |  | Others (Specify) | | 4 | |  |
|  |  | Don’t know/No reason | | 77 | |  |
| **EXPANDED: Diet** | | | | | | |
| 3015 | What type of **oil or fat is most often** used for meal preparation in your household?  *SELECT ONLY ONE* | Vegetable oil | | 1 | |  |
|  |  | Lard or suet | | 2 | |  |
|  |  | Butter or ghee | | 3 | |  |
|  |  | Margarine | | 4 | |  |
|  |  | Other | | 5 | |  |
|  |  | None in particular | | 6 | |  |
|  |  | None used | | 7 | |  |
|  |  | Don’t know | | 77 | |  |
|  |  | Other (Specify) | | └─┴─┴─┴─┴─┴ | |  |
| 3016 | On average, how many meals per week do you eat that were not prepared at your home? **Note**: Meal means **breakfast**, **lunch** or **dinner**. | Number | | ─┴─┘ | |  |
|  |  | Don’t know | | 77 | |  |
| 3017 | **Number** of beverages consumed in a day for the past 7 days (TOTAL NUMBER OF DRINKS) ***1 standard drink is roughly equivalent to 340 ml** of sugar based fluid in a can, bottle or glass)  **Attempt all** | 100% fruit juice | | | 🞏🞏 |  |
|  |  | Fizzy or soft drink | | | 🞏🞏 |  |
|  |  | Regular soda or pop | | | 🞏🞏 |  |
|  |  | Diet pop/Diet soda | | | 🞏🞏 |  |
|  |  | Regular sports drinks/Energy drinks | | | 🞏🞏 |  |
|  |  | Fruit nectar | | | 🞏🞏 |  |
|  |  | Don’t know | | | 77 |  |
| 3018 | **Number** of cups/mugs of hot drinks with 4 tsp of sugar taken in a day  (1 CUP/MUG is ≠ 150-200ml capacity) | Coffee | | | 🞏🞏 |  |
|  |  | Tea | | | 🞏🞏 |  |
| 3019 | How often is **salt or salty sauce** added to your daily meal right before you eat or as you are eating? | Always | | | 1 |  |
|  |  | Often | | | 2 |  |
|  |  | Sometimes | | | 3 |  |
|  |  | Rarely | | | 4 |  |
|  |  | Never | | | 5 |  |
|  |  | Don’t know | | | 77 |  |
| 3020 | How often is **salt added** in cooking or preparing foods in your household? | Always | | | 1 |  |
|  |  | Often | | | 2 |  |
|  |  | Sometimes | | | 3 |  |
|  |  | Rarely | | | 4 |  |
|  |  | Never | | | 5 |  |
|  |  | Don’t know | | | 77 |  |
| 3021 | Do you think that **lowering salt** in your diet is important? | Yes very important | | | 1 |  |
|  |  | Yes somewhat important | | | 2 |  |
|  |  | Not at all important | | | 3 |  |
|  |  | Don't know | | | 77 |  |
| 3022 | Do you think that too much salt in your diet could cause a serious **health problem**? | Yes | | | 1 |  |
|  |  | No | | | 2 |  |
|  |  | Don't know | | | 77 |  |
| **Physical Activity -** Now I am going to ask you about the time you spent being physically active in the last 7 days. Please answer each question even if you do not consider yourself to be an active person. Think about the activities you do at work, as part of your house and yard work, to get from places to place, and in your spare time for recreation, exercise or sport. | | | | | | |
| 3023 | **Vigorous Activity:** Now, think about all the vigorous activities which take hard physical effort that you did in the last 7 days. Vigorous activities make you breathe much harder than normal and may include heavy lifting, digging, aerobics, or fast bicycling. Think only about those physical activities that you did for at least 10 minutes at a time. During the last 7 days, on **how** **many days** did you do vigorous physical activities? | Number Of Days: | ***└─┴─┘*** | | | **If none, go to Q3026** |
| 3024 | Does your work involve **vigorous-intensity activity** that causes large increases in breathing or heart rate like *[carrying or lifting* *heavy loads, digging or construction work]* for at least 10 minutes continuously?  *INSERT EXAMPLES* | Yes | 1 | | |  |
|  |  | No | 2 | | |  |
| 3025 | How much time did you usually spend doing **vigorous physical activities** on one of those days? | Hours per day | ______ | | |  |
|  |  | Minutes per day | ______ | | |  |
| 3026 | Does your work involve **moderate-intensity activity**, that causes small increases in breathing or heart rate such as brisk walking *[or carrying light loads]* for at least 10 minutes continuously?  *INSERT EXAMPLES* | Yes | 1 | | |  |
|  |  | No | 2 | | |  |
| 3027 | Now think about activities which take moderate physical effort that you did in the last 7 days. Moderate physical activities make you breathe somewhat harder than normal and may include carrying light loads, bicycling at a regular pace, or doubles tennis. Do not include walking. Again, think about only those physical activities that you did for at least 10 minutes at a time. During the last 7 days, on **how many days** did you do moderate physical activities? |  | | | | ***If none, go to Q3030*** |
| 3028 | How much time did you usually spend doing **moderate physical activities** on one of those days? | Hours per day | ______ | | |  |
|  |  | Minutes per day | ______ | | |  |

| 3029 | Do you **walk or use a bicycle** *(pedal cycle)* for at least 10 minutes continuously to get to and from places? | Yes | 1 | |  |
| --- | --- | --- | --- | --- | --- |
|  |  | No | 2 | |  |
| 3030 | **Walking:** Now think about the time you spent walking in the last 7 days. This includes at work and at home, walking to travel from place to place, and any other walking that you might do solely for recreation, sport, exercise, or leisure. During the last 7 days, on **how many days** did you walk for at least 10 minutes at a time? |  |  | | ***If none, go to Q3032*** |
| 3031 | How much time did you usually spend walking on one of those days? | Hours per day | _____ | |  |
|  |  | Minutes per day | ______ | |  |
| **Environmental risk factors - water and sanitation** | | | | | |
| 3032 | What type of floor does your dwelling / house have? | Hard floor (tile, cement, brick, wood) | | 1 |  |
|  |  | Earth floor | | 2 |  |
| 3033 | What type of wall does your dwelling / house have? | Cement, brick, stone or wood | | 1 |  |
|  |  | Mud brick | | 2 |  |
|  |  | Thatch and other | | 3 |  |
|  |  | Plastic sheet | | 4 |  |
|  |  | Metal sheet | | 5 |  |
|  |  | Other | | 6 |  |
| 3034 | What is the main source of drinking water for members of this household? | Piped water through house connection or yard | | 1 | ***If 1, go to Q3036*** |
|  |  | Public standpipe | | 2 |  |
|  |  | Protected tube well or bore hole | | 3 |  |
|  |  | Protected dug well or protected spring | | 4 |  |
|  |  | Unprotected dug well or spring | | 5 |  |
|  |  | Rainwater (into tank or cistern) | | 6 |  |
|  |  | Water taken directly from pond-water or stream | | 7 |  |
|  |  | Tanker-truck, vendor | | 8 |  |
| 3035 | How long does it take to get there, get water and come back? | Less than 5 minutes | | 1 |  |
|  |  | Between 5 to 30 minutes | | 2 |  |
|  |  | Between 30 to 60 minutes | | 3 |  |
|  |  | Between 60 to 90 minutes | | 4 |  |
|  |  | More than 90 minutes | | 5 |  |
| 3036 | Are there at least 20 litres of water per person (about one bucket) available per day (for drinking, cooking, personal hygiene etc.) in the household? | Yes | | 1 |  |
|  |  | No | | 2 |  |
| 3037 | What type of toilet facilities does your household use? | Flush to piped sewage system | | 1 |  |
|  |  | Flush to septic tank | | 2 |  |
|  |  | Pour flush latrine | | 3 |  |
|  |  | Covered dry latrine (with privacy) | | 4 |  |
|  |  | Uncovered dry latrine (without privacy) | | 5 |  |
|  |  | Bucket latrine (where fresh excreta are manually removed) | | 6 |  |
|  |  | No facilities (open defecation) | | 7 |  |
|  |  | Other | | 8 |  |

| 3038 | How far is the facility from your dwelling/house? | Within property/yard, used by single household | 1 |  |
| --- | --- | --- | --- | --- |
|  |  | Within property/yard, used by multiple household | 2 |  |
|  |  | Outside property/yard, private | 3 |  |
|  |  | Outside property/yard, shared | 4 |  |
| 3039 | What type of fuel does your household mainly use for cooking? | Gas | 1 | ***If 1 and 2, go to Q3042*** |
|  |  | Electricity | 2 |  |
|  |  | Kerosene | 3 |  |
|  |  | Coal | 4 |  |
|  |  | Charcoal | 5 |  |
|  |  | Wood | 6 |  |
|  |  | Agriculture/crop | 7 |  |
|  |  | Animal dung | 8 |  |
|  |  | Shrubs/grass | 9 |  |
|  |  | Other | 10 |  |
| 3040 | What type of cooking stove is used in your house? | Open fire or stove without chimney or hood | 1 |  |
|  |  | Open fire or stove with chimney or hood | 2 |  |
|  |  | Closed stove with chimney | 3 |  |
|  |  | Other | 4 |  |
| 3041 | Where is cooking usually done? | In a room used for living or sleeping | 1 |  |
|  |  | In a separate room used as kitchen | 2 |  |
|  |  | In a separate building used as kitchen | 3 |  |
|  |  | Outdoors | 4 |  |
| 3042 | Do you heat your house when it is cold? | Yes | 1 | ***If no, go to Q4001*** |
|  |  | No | 2 |  |
| 3043 | What type of fuel does your household mainly use for heating? | Gas | 1 | ***If 1 and 2, go to Q4001*** |
|  |  | Electricity | 2 |  |
|  |  | Kerosene | 3 |  |
|  |  | Coal | 4 |  |
|  |  | Charcoal | 5 |  |
|  |  | Wood | 6 |  |
|  |  | Agriculture/crop | 7 |  |
|  |  | Animal dung | 8 |  |
|  |  | Shrubs/grass | 9 |  |
|  |  | Other | 10 |  |
| 3044 | What type of heating stove is used in your house? | Open fire or stove without chimney or hood | 1 |  |
|  |  | Open fire or stove with chimney or hood | 2 |  |
|  |  | Closed stove with chimney | 3 |  |
|  |  | Other | 4 |  |

| **SECTION 4: CHRONIC NON COMMUNACABLE DISEASES** |
| --- |

| ***READ TO RESPONDENTS***: Now I would like to read to you questions about some health problems or health care needs that you and the young children in this house may have experienced, and the treatment or medical care that you may have received. | | | | | | |
| --- | --- | --- | --- | --- | --- | --- |
| **ATHRITIS** | | | | | | |
| 4001 | Have you ever been diagnosed with **arthritis** (a disease of the joints)? | Yes | 1 | | | ***If no, go to Q4003*** |
|  |  | No | 2 | | |  |
|  |  | Don’t know | 8 | | |  |
| 4001a | Have you ever been treated for it? | Yes | 1 | | | ***Refer to question Q4001*** |
|  |  | No | 2 | | |  |
|  |  | Don’t know | 8 | | |  |
| 4002 | Have you been taking any medications or other treatment for it during the last 2 weeks? | Yes | 1 | | | ***Only ask this question if the answer is ‘yes’ in Q4001*** |
|  |  | No | 2 | | |  |
|  |  | Don’t know | 8 | | |  |
| During the last 12 months have you experienced any of the following? | | | | | | |
| 4003 | Pain, aching, stiffness or swelling in or around the joint (like arms, hands, legs or feet) which were not related to an injury and lasted for more than a month? | Yes | 1 | | ***If no, go to Q4008*** | |
|  |  | No | 2 | |  |  |
|  |  | Don’t know | 8 | |  |  |
| 4004 | Stiffness in the joint in the morning after getting up from bed, or after a long rest of the joint without movement? | Yes | 1 | | ***If Q4003 and Q4004 both ‘No’ skip to Q4008*** | |
|  |  | No | 2 | |  |  |
|  |  | Don’t know | 8 | |  |  |
| 4005 | How long does this stiffness last?  *READ CHOICES AND MARK AS APPROPRIATE* | About 30 mins or less | 1 | |  | |
|  |  | More than 30 mins | 2 | |  |  |
| 4006 | Does this stiffness go away after exercise or movement in the joint? | Yes | 1 | |  | |
|  |  | No | 2 | |  |  |
| 4008 | Have you experienced back pain (including disc problems) during the last 30 days? | Yes | 1 | | ***If no, go to Q4010*** | |
|  |  | No | 2 | |  |  |
| 4009 | How many days did you have this back pain during the last 30 days? | _______ |  | |  | |
| **STROKE** | | | | | | |
| 4010 | Have you ever been told by a health professional that you have had a stroke? | Yes | 1 | ***If no, go to Q4014*** | | |
|  |  | No | 2 |  |  |  |
|  |  | Don’t know | 8 |  |  |  |
| 4011 | Have you been taking any medications or other treatment for it…? | | |  | | |
|  | 1. During the last 2 weeks? | Yes | 1 |  |  |  |
|  |  | No | 2 |  |  |  |
|  | 1. During the last 12 months | Yes | 1 |  |  |  |
|  |  | No | 2 |  |  |  |
| 4012 | Have you ever suffered from sudden onset of paralysis or weakness in your arms or legs on one side of your body for more than 24 hours? | Yes | 1 |  | | |
|  |  | No | 2 |  |  |  |
|  |  | No | 2 |  |  |  |

| **ANGINA** | | | | | | | |
| --- | --- | --- | --- | --- | --- | --- | --- |
| 4014a | Have you ever been diagnosed with **angina or angina pectoris (a type of heart disease)?** | | Yes | | 1 | | ***If no, go to Q4016*** |
|  |  |  | No | | 2 | |  |
|  |  |  | Don’t Know | | 8 | |  |
| 4014b | Have you ever been treated for it? | | Yes | | 1 | |  |
|  |  |  | No | | 2 | |  |
|  |  |  | Don’t Know | | 8 | |  |
| 4015 | Have you been taking any medications or other treatment for it during the last 2 weeks? | | Yes | | 1 | |  |
|  |  |  | No | | 2 | |  |
|  |  |  | Don’t Know | | 8 | |  |
| During the last 12 months, have you experienced any of the following: | | | | | | | |
| 4016 | Pain or discomfort in your chest when you walk uphill or hurry? | Yes | | | 1 |  | |
|  |  | No | | | 2 |  |  |
|  |  | Never walk uphill | | | 3 |  |  |
| 4017 | Pain or discomfort in your chest when you walk at an ordinary pace on level ground? | Yes | | | 1 | ***If no, go to Q4022*** | |
|  |  | No | | | 2 |  |  |
| 4018 | What do you do if you get the pain or discomfort when you are walking? *READ CHOICES* | Stop or slow down | | | 1 |  | |
|  |  | Carry on after taking a pain relieving medicine that dissolves in your mouth | | | 2 |  |  |
|  |  | Carry on | | | 3 |  |  |
| 4019 | If you stand still, what happens to the pain or discomfort? *READ CHOICES* | Relieved | | | 1 |  | |
|  |  | Not relieved | | | 2 |  |  |
| 4020 | Will you show me where you usually experience the pain or discomfort?  *RECORD ALL AREAS OF BODY MENTIONED OR SHOWED* | Upper or middle chest | | | 1 |  | |
|  |  | Lower chest | | | 2 |  |  |
|  |  | Left arm | | | 3 |  |  |
|  |  | Other | | | 4 |  |  |
| **DIABETES** | | | | | | | |
| 4021 | Have you ever been diagnosed with diabetes (high blood sugar)? | Yes | | 1 | | ***If no, go to Q4025*** | |
|  |  | No | | 2 | |  |  |
|  |  | Don’t Know | | 8 | |  |  |
| 4022 | Have you ever been treated for it? | Yes | | 1 | |  | |
|  |  | No | | 2 | |  |  |
|  |  | Don’t Know | | 8 | |  |  |
| 4023 | Have you been taking insulin or other blood sugar lowering medications in the last 2 weeks? | Yes | | 1 | |  | |
|  |  | No | | 2 | |  |  |
|  |  | Don’t Know | | 8 | |  |  |
| 4024 | Are you following a special diet, exercise regime or weight control program for diabetes | Yes | | 1 | |  | |
|  |  | No | | 2 | |  |  |
|  |  | Don’t know | | 3 | |  |  |

| **CHRONIC LUNG DISEASE** | | | | | | | | | | | | | | |  |  |  |  |
| --- | --- | --- | --- | --- | --- | --- | --- | --- | --- | --- | --- | --- | --- | --- | --- | --- | --- | --- |
| 4025 | Have you ever been diagnosed with chronic lung disease (emphysema, bronchitis, COPD)? | | | | Yes | | | | 1 | | ***If no, go to Q4027*** | | | |  |  |  |  |
|  |  |  |  |  | No | | | | 2 | |  |  |  |  |  |  |  |  |
| 4026 | Have you been taking any medications or treatment in the last 2 weeks? | | | | Yes | | | | 1 | |  | | | |  |  |  |  |
|  |  |  |  |  | No | | | | 2 | |  |  |  |  |  |  |  |  |
|  | …in the last 12 months? | | | | Yes | | | | 1 | |  |  |  |  |  |  |  |  |
|  |  |  |  |  | No | | | | 2 | |  |  |  |  |  |  |  |  |
| 4027 | During the last 12 months, have you experienced any shortness of breath at rest? *(while awake)* | | | | Yes | | | | 1 | |  | | | |  |  |  |  |
|  |  |  |  |  | No | | | | 2 | |  |  |  |  |  |  |  |  |
| 4028 | During the last 12 months, have you experienced any coughing or wheezing for ten minutes or more at a time? | | | | Yes | | | | 1 | |  | | | |  |  |  |  |
|  |  |  |  |  | No | | | | 2 | |  |  |  |  |  |  |  |  |
| 4029 | During the last 12 months, have you experienced any coughing up sputum or phlegm for most days of the month for at least 3 months? | | | | Yes | | | | 1 | |  | | | |  |  |  |  |
|  |  |  |  |  | No | | | | 2 | |  |  |  |  |  |  |  |  |
| **ASTHMA** | | | | | | | | | | | | | | |  |  |  |  |
| 4030 | Have you ever been diagnosed with **asthma (an allergic respiratory disease)?** | | | Yes | | | | 1 | | | | | ***If no, go to Q4033*** | |  |  |  |  |
|  |  |  |  | No | | | | 2 | | | | |  |  |  |  |  |  |
|  |  |  |  | Don’t Know | | | | 8 | | | | |  |  |  |  |  |  |
| 4031 | Have you ever been treated for it? | | | Yes | | | | 1 | | | | |  | |  |  |  |  |
|  |  |  |  | No | | | | 2 | | | | |  |  |  |  |  |  |
|  |  |  |  | Don’t Know | | | | 8 | | | | |  |  |  |  |  |  |
| 4032 | Have you been taking any medications or other treatment for it during the last 2 weeks? | | | Yes | | | | 1 | | | | |  | |  |  |  |  |
|  |  |  |  | No | | | | 2 | | | | |  |  |  |  |  |  |
|  |  |  |  | Don’t Know | | | | 8 | | | | |  |  |  |  |  |  |
| During the last 12 months, have you experienced any of the following: | | | | | | | | | | | | | | |  |  |  |  |
| 4033 | Attacks of wheezing or whistling breathing? | | | Yes | | | | 1 | | | | | ***If no, go to Q4040*** | |  |  |  |  |
|  |  |  |  | No | | | | 2 | | | | |  |  |  |  |  |  |
| 4036 | Attack of wheezing that came on after you stopped exercising or some other physical activity? | | | Yes | | | | 1 | | | | |  | |  |  |  |  |
|  |  |  |  | No | | | | 2 | | | | |  |  |  |  |  |  |
| 4037 | A feeling of tightness in your chest? | | | Yes | | | | 1 | | | | |  | |  |  |  |  |
|  |  |  |  | No | | | | 2 | | | | |  |  |  |  |  |  |
| 4038 | Have you woken up with a feeling of tightness in your chest in the morning or any other time? | | | Yes | | | | 1 | | | | |  | |  |  |  |  |
|  |  |  |  | No | | | | 2 | | | | |  |  |  |  |  |  |
| 4039 | Have you had an attack of shortness of breath that came on without obvious cause when you were not exercising or doing some physical activity? | | | Yes | | | | 1 | | | | |  | |  |  |  |  |
|  |  |  |  | No | | | | 2 | | | | |  |  |  |  |  |  |
| **HYPERTENSION** | | | | | | | | | | | | | | |  |  |  |  |
| 4040 | Have you ever been diagnosed with hypertension (high blood pressure? | | | Yes | | | | 1 | | | ***If no, go to Q4043*** | | | |  |  |  |  |
|  |  |  |  | No | | | | 2 | | |  |  |  |  |  |  |  |  |
| 4041 | Have you ever been treated for it? | | | Yes | | | | 1 | | |  | | | |  |  |  |  |
|  |  |  |  | No | | | | 2 | | |  |  |  |  |  |  |  |  |
|  |  |  |  | Don’t Know | | | | 8 | | |  |  |  |  |  |  |  |  |
| 4042 | Have you been taking any medications or other treatment for it during the last 2 weeks? | | | Yes | | | | 1 | | |  | | | |  |  |  |  |
|  |  |  |  | No | | | | 2 | | |  |  |  |  |  |  |  |  |
|  |  |  |  | Don’t Know | | | | 8 | | |  |  |  |  |  |  |  |  |
| **CERVICAL CANCER AND BREAST CANCER SCREENING (WOMEN ONLY)** | | | | | | | | | | | | | | |  |  |  |  |
| 4043 | When was the last time you had a pelvic examination, if ever?  (By pelvic examination, I mean when a doctor or nurse examined your vagina and uterus?)  *ENTER "00" IF LESS THAN 1 YEAR AGO.* | | _______ | | | Years ago | | | | | |  | | |  |  |  |  |
|  |  |  | Less than a year ago | | | Enter 00 | | | | | |  |  |  |  |  |  |  |
|  |  |  | Never had exam | | | 98 | | | | | |  |  |  |  |  |  |  |
| 4044 | The last time you had the pelvic examination; did you have a PAP smear test?  (By PAP smear test, I mean did a doctor or nurse use a swab or stick to wipe from inside your vagina, take a sample and send it to a laboratory?) | | Yes | | | 1 | | | | | | ***If no, go to Q4046*** | | |  |  |  |  |
|  |  |  | No | | | 2 | | | | | |  |  |  |  |  |  |  |
| 4045 | When was the last time you had a mammography, if ever?  (That is, an x-ray of your breasts taken to detect breast cancer at an early stage.) | | _____ | | | Years ago | | | | | |  | | |  |  |  |  |
|  |  |  | Less than a year ago | | | Enter 00 | | | | | |  |  |  |  |  |  |  |
|  |  |  | Never had exam | | |  | | | | | |  |  |  |  |  |  |  |
| **OTHER DISEASES** | | | | | | | | | | | | | | |  |  |  | No |
| 4046 | During the past 12 months have you been told by a doctor or other health workers that you have/had or suffered from the following problems/conditions? | Eye/vision problem( like Cataract retinopathy) | | | | | Yes | | | No | | | |  |  |  |  |  |
|  |  | Nerves problem | | | | | Yes | | | No | | | |  |  |  |  |  |
|  |  | Kidney problem | | | | | Yes | | | No | | | |  |  |  |  |  |
|  |  | Skin problem | | | | | Yes | | | No | | | |  |  |  |  |  |
|  |  | Mental Illness  (Such as depression, loneliness, suicidal attempt, no close friends etc.) | | | | | Yes | | | No | | | |  |  |  |  |  |
|  |  | Cancer | | | | | Yes | | | No | | | |  |  |  |  |  |
|  |  | Any other conditions  Specify__________________ | | | | | Yes | | | No | | | |  |  |  |  |  |
| 4047 | These symptoms that you said you experienced in the last 12 months, have you experienced them in the last 2 weeks? | Eye/vision Problem ( like Cataract retinopathy) | | | | | Yes | | | No | | | |  |  |  |  |  |
|  |  | Nerves problem | | | | | Yes | | | No | | | |  |  |  |  |  |
|  |  | Kidney problem | | | | | Yes | | | No | | | |  |  |  |  |  |
|  |  | Skin problem | | | | | Yes | | | No | | | |  |  |  |  |  |
|  |  | Mental Illness  (Such as depression, loneliness, suicidal attempt, no close friends etc.) | | | | | Yes | | | No | | | |  |  |  |  |  |
|  |  | Cancer | | | | | Yes | | |  | | | |  |  |  |  |  |

| **SECTION 5-HEALTH CARE UTILIZATION AND EXPENDITURE**  I would now like to know about your recent experiences with obtaining health care from health care workers, hospitals, clinics and the health care system. I want to know if you needed health care recently, and if so, why you needed health care and what type of health care provider you received care from. | | | | | | | | | | | | | | |
| --- | --- | --- | --- | --- | --- | --- | --- | --- | --- | --- | --- | --- | --- | --- |
| 5001 | When was the last time that you needed health care?  *INTERVIEWER: this can be inpatient or outpatient care. If less than one month ago, enter “00” for years and "00" for months.* | | | | | _________ | | Years ago | | | | | ***If more than 3 years ago, go to Q6001*** | |
|  |  |  |  |  |  | _________ | | Months ago | | | | |  |  |
|  |  |  |  |  |  | Never | | 98 | | | | | ***If never, go to Q6001*** | |
|  |  |  |  |  |  | Don’t know | | 8 | | | | |  | |
|  | **5001(a)** *If 'don’t know'*, Was it more than 3 years ago? | | | | | Yes | | 1 | | | | | ***If yes, go to Q6001*** | |
|  |  |  |  |  |  | No | | 2 | | | | |  | |
| 5002 | The last time you needed health care, did you get health care? | | | | | Yes | | 1 | | | | |  | |
|  |  |  |  |  |  | No | | 2 | | | | |  | |
| 5002a | What was the main reason you needed care, even if you did not get care?  *INTERVIEWER: Respondent can select ONLY one main reason for visit. USE SHOWCARD (APPENDIX RESPONSE SCALES)* | | | Chronic pain in your joints/arthritis (joints, back, neck) | | | | | | | 1 | |  | |
|  |  |  |  | Diabetes or related complications | | | | | | | 2 | |  |  |
|  |  |  |  | Problems with your heart including unexplained pain in chest (angina) | | | | | | | 3 | |  |  |
|  |  |  |  | Problems with your breathing (asthma) | | | | | | | 4 | |  |  |
|  |  |  |  | High blood pressure/hypertension | | | | | | | 5 | |  |  |
|  |  |  |  | Stroke/sudden paralysis of one side of body | | | | | | | 6 | |  |  |
|  |  |  |  | Cancer | | | | | | | 7 | |  |  |
|  |  |  |  | Nutritional deficiencies | | | | | | | 8 | |  |  |
|  |  |  |  | Chronic lung disease | | | | | | | 9 | |  |  |
|  |  |  |  | Communicable disease | | | | | | | 10 | |  |  |
|  |  |  |  | Other (specify) | | | | | | | 87 | |  |  |
| 5003 | Which reason(s) best explains why you did not get health care?  *INTERVIEWER: Circle all that the respondent indicates.* | | | Could not afford the cost of the visit | | | | | | | 1 | |  |  |
|  |  |  |  | No transport available | | | | | | | 2 | |  |  |
|  |  |  |  | Could not afford the cost of transport | | | | | | | 3 | |  |  |
|  |  |  |  | You were previously badly treated | | | | | | | 4 | |  |  |
|  |  |  |  | Could not take time off work or had other commitments | | | | | | | 5 | |  |  |
|  |  |  |  | The health care provider's drugs or equipment were inadequate | | | | | | | 6 | |  |  |
|  |  |  |  | The health care provider's skills were inadequate | | | | | | | 7 | |  |  |
|  |  |  |  | You did not know where to go | | | | | | | 8 | |  |  |
|  |  |  |  | You tried but were denied health care | | | | | | | 9 | |  |  |
|  |  |  |  | You thought you were not sick enough | | | | | | | 10 | |  |  |
|  |  |  |  | Other(specify) | | | | | | | 87 | |  |  |
| 5004 | Thinking about health care you needed in the last 3 years, where did you go most often when you felt sick or needed to consult someone about your health?  *INTERVIEWE****R:*** *Only one answer allowed.* | | | Private doctor’s office | | | | | | | 1 | |  | |
|  |  |  |  | Private clinic or health care facility | | | | | | | 2 | |  |  |
|  |  |  |  | Private hospital | | | | | | | 3 | |  |  |
|  |  |  |  | Public clinic or health care facility | | | | | | | 4 | |  |  |
|  |  |  |  | Public hospital | | | | | | | 5 | |  |  |
|  |  |  |  | Charity or church run clinic | | | | | | | 6 | |  |  |
|  |  |  |  | Charity or church run hospital | | | | | | | 7 | |  |  |
|  |  |  |  | Traditional healer [*use local term*] | | | | | | | 8 | |  |  |
|  |  |  |  | Pharmacy or dispensary | | | | | | | 9 | |  |  |
|  |  |  |  | Other(specify) | | | | | | | 87 | |  |  |
| **Inpatient hospital care**  The next two questions ask about any overnight stay in a hospital or other health care facility you have had in the last 12 months. | | | | | | | | | | | | | | |
| 5005 | In the last 12 months, have you ever stayed overnight in a hospital or long-term care facility? | | Yes, a hospital | | | | 1 | | | | | |  | |
|  |  |  | Yes, long term care facility | | | | 2 | | | | | |  |  |
|  |  |  | Both ( hospital and long term care facility) | | | | 3 | | | | | |  |  |
|  |  |  | No | | | | 4 | | | | | |  |  |
| 5006 | When was the last overnight stay in a hospital or long term care facility?  *INTERVIEWER: If less than one month ago, enter “00” for years and "00" for months.* | | ____years ago | | | | ___months ago | | | | | |  | |
|  |  |  | Don’t know | | | | -8 | | | | | |  |  |
| Now I would like to know about more recent times - if you've had any overnight stays in a hospital or other type of health care facility in the last 12 months. | | | | | | | | | | | | | | |
| 5007 | Over the last 12 months, how many different times were you a patient in a hospital/long-term care facility for at least one night? | | | | | _____ times |  | | | ***IF "00" (NO OVERNIGHT STAYS), go to Q5026*** | | | | |
|  |  |  |  |  |  | Don’t know | -8 | | |  |  |  |  |  |
| I want to know more about why you needed an overnight stay in a health care facility. Starting with the most recent stay, I want to know more about your overnight stays, including why you needed an overnight stay each time. But first I would like you to come back to thinking about your last overnight hospital stay only. | | | | | | | | | | | | | | |
| 5008a | What type of hospital or facility was it? Remember we are asking now about your last (most recent) overnight stay. | Public hospital | | | | | | | 1 | | |  | | |
|  |  | Private hospital | | | | | | | 2 | | |  |  |  |
|  |  | Charity or church-run hospital | | | | | | | 3 | | |  |  |  |
|  |  | Old person's home or long-term care facility | | | | | | | 4 | | |  |  |  |
|  |  | Other(specify) | | | | | | | 7 | | |  |  |  |
|  | 1. What was the name of this hospital or facility? | ____________________________________________ | | | | | | | | | |  |  |  |
| 5008b | Which reason best describes why you were last hospitalized?  *INTERVIEWER: Respondent can select only ONE main reason for visit.* | Chronic pain in your joints/arthritis (joints, back, neck) | | | | | | | 1 | | |  | | |
|  |  | Diabetes or related complications | | | | | | | 2 | | |  |  |  |
|  |  | Problems with your heart including unexplained pain in chest (angina) | | | | | | | 3 | | |  |  |  |
|  |  | Problems with your breathing (asthma) | | | | | | | 4 | | |  |  |  |
|  |  | High blood pressure /hypertension | | | | | | | 5 | | |  |  |  |
|  |  | Stroke/sudden paralysis of one side of body | | | | | | | 6 | | |  |  |  |
|  |  | Cancer | | | | | | | 7 | | |  |  |  |
|  |  | Chronic lung disease | | | | | | |  | | |  |  |  |
|  |  | Communicable diseases | | | | | | |  | | |  |  |  |
|  |  | Other (specify) | | | | | | | 8 | | |  |  |  |
| 5009a | How did you get there?  *INTERVIEWER: Circle all that the respondent mentions.* | Private vehicle | | | | | | | 1 | | |  | | |
|  |  | Public transportation | | | | | | | 2 | | |  |  |  |
|  |  | Taxicab | | | | | | | 3 | | |  |  |  |
|  |  | Ambulance or emergency vehicle | | | | | | | 4 | | |  |  |  |
|  |  | Bicycle | | | | | | | 5 | | |  |  |  |
|  |  | Walked | | | | | | | 6 | | |  |  |  |
|  |  | *DON'T KNOW* | | | | | | | 8 | | |  |  |  |
| 5009b | About how long did it take you to get there? | | | | *______*hours | | ________minutes | | | | | | |  |
|  |  |  |  |  | *Don’t know* | | -8 | | | | | | |  |
| 5010 | Who paid for this hospitalization? Anyone else?  *INTERVIEWER: Circle all responses. Probe to see if anyone else paid or contributed to paying for the care?* | | | | Respondent | | 1 | | | | | | |  |
|  |  |  |  |  | Spouse/partner | | 2 | | | | | | |  |
|  |  |  |  |  | Son/daughter | | 3 | | | | | | |  |
|  |  |  |  |  | Other family member | | 4 | | | | | | |  |
|  |  |  |  |  | Non-family member | | 5 | | | | | | |  |
|  |  |  |  |  | Mandatory insurance scheme | | 6 | | | | | | |  |
|  |  |  |  |  | Voluntary insurance scheme | | 7 | | | | | | |  |
|  |  |  |  |  | Hospitalization was free | | 8 | | | | | | |  |

| 5011 | Thinking about your last [hospital] stay, how much did you or your family/household members pay out-of- pocket for: | | 1. Health care provider's fees | | | | ____________ | | | |  | |  |
| --- | --- | --- | --- | --- | --- | --- | --- | --- | --- | --- | --- | --- | --- |
|  |  |  | 1. Medicines | | | | ____________ | | | |  |  |  |
|  |  |  | 1. Tests | | | | ___________ | | | |  |  |  |
|  |  |  | 1. Transport | | | | ____________ | | | |  |  |  |
|  |  |  | 1. Other specify | | | | ____________ | | | |  |  |  |
| 5012 | About how much in total did you or a family/household member pay out-of-pocket for this hospitalization? | | ______________ | | | |  | | | |  | |  |
| 5013 | Overall, how satisfied were you with the care you received during your last [hospital] stay? | | Very satisfied | | | | | 1 | | |  | |  |
|  |  |  | Satisfied | | | | | 2 | | |  |  |  |
|  |  |  | Neither satisfied nor dissatisfied | | | | | 3 | | |  |  |  |
|  |  |  | Dissatisfied | | | | | 4 | | |  |  |  |
|  |  |  | Very dissatisfied | | | | | 5 | | |  |  |  |
| 5014 | What was the outcome or result of your visit to the [hospital]? Did your condition… | | Get much better | | | | | 1 | | |  | |  |
|  |  |  | Get better | | | | | 2 | | |  |  |  |
|  |  |  | No change | | | | | 3 | | |  |  |  |
|  |  |  | Get worse | | | | | 4 | | |  |  |  |
|  |  |  | Get much worse | | | | | 5 | | |  |  |  |
| 5015 | Was this the outcome or result you had expected? | | | | Yes | | | 1 | | |  | |  |
|  |  |  |  |  | No | | | 2 | | |  |  |  |
| **OUTPATIENT CARE AND CARE**  Now I will shift away from questions about overnight stays – to questions about health care you received that did not include an overnight hospital stay. The following questions are about care you received at a hospital, health centre, clinic, private office or at home from a health care worker, but where you did not stay overnight. | | | | | | | | | | | | |  |
| 5026 | Over the last 12 months, did you receive any care, health care NOT including an overnight stay in hospital or long-term care Facility_________________________________________ | | | | | Yes | | | 1 | | ***If no, go to Q6001*** | |  |
|  |  |  |  |  |  | No | | | 2 | |  |  |  |
| 5027 | In total, how many times did you receive health Care or consultation in the last 12 months? | | | | | ______________ | | | | |  | |  |
| Now I would like you to think about the most recent visit - and will ask you specifically about your last or most recent visit. | | | | | | | | | | | | |  |
| 5028a | What was the last (most recent) health care Facility you visited in the last 12 months?  ***INTERVIEWER:*** *read out responses, circle one option only*  *Other, specify:________________* | 1. Home visit 2. Private clinic or health care facility 3. Private hospital 4. Public clinic or health care facility 5. Public hospital 6. Charity or church run clinic 7. Charity or church run hospital 8. Private doctor’s office | | | | | | | | | |  |  |
| 5028b | What was the name of this health care facility or provider? | ______________________________ | | | | | | | | | |  |  |
| 5029a | Which was the last (most recent) health care provider you visited? | 1. Medical Doctor (Including Surgeon Gynaecologist, Psychiatrist, Ophthalmologist) 2. Nurse/Midwife 3. Dentist 4. Physiotherapist Or Chiropractor 5. Traditional Medicine Practitioner (Use Local Name) 6. Pharmacist, Druggist 7. Home Health Care Worker 8. *Don't Know* | | | | | | | | | |  |  |
| 5029b | A chronic (on-going) condition, new condition, both**.** Was this visit **for** routine check-up, ongoing chronic condition, new or both? | 1. Routine Check-Up 2. New 3. Both 4. Ongoing Chronic | | | | | | | | | |  |  |
| 5029c | Which reason best describes why you needed this visit?  *INTERVIEWER: Respondent can select only ONE main reason for visit* | 1. Diabetes or related complications 2. Problems with your heart including unexplained pain in chest 3. Problems with your mouth, teeth or swallowing 4. Problems with your breathing 5. High blood pressure /hypertension 6. Stroke/sudden paralysis of one side of body 7. Generalized pain (stomach, muscle or other nonspecific pain) 8. Depression or anxiety 9. Cancer 10. Other, specify | | | | | | | | | |  |  |
| 5029d | Thinking about your last visit, how did you get there?  *INTERVIEWER: Circle all that the respondent mentions.* | 1. Private vehicle | | | | | | | | | |  |  |
|  |  | 1. Public transportation | | | | | | | | | |  |  |
|  |  | 1. Taxi/cab | | | | | | | | | |  |  |
|  |  | 1. Ambulance or emergency vehicle | | | | | | | | | |  |  |
|  |  | 1. Bicycle | | | | | | | | | |  |  |
|  |  | 1. Walked | | | | | | | | | |  |  |
|  |  | 1. Don’t know | | | | | | | | | |  |  |
|  |  | 1. Not applicable | | | | | | | | | |  |  |
| 5029e | About how long did it take you to get there? | HOURS: MINUTES ____________:____________  8. *DON'T KNOW* | | | | | | | | | |  |  |
| 5029f | Who paid for this most recent visit?  *INTERVIEWER: circle all responses. Probe to see if* *anyone else paid or contributed to paying for the care?* | 1. Respondent 2. Spouse/Partner 3. Son/Daughter 4. Other Family Member 5. Non-Family Member 6. Mandatory Insurance Scheme 7. Voluntary Insurance Scheme 8. It Was Free …………………………… | | | | | | | | | |  |  |
| 5029g | Thinking about your last visit, how much did you or your household pay for:  *INTERVIEWER: Only write "0" if the service was free, If a person did not have tests or drugs, enter 99998 for “Not applicable, did not have* *(local currency)* | | | 1. Health Care Provider's] Fees 2. Medicines 3. Tests 4. Transport 5. Other, Specify | | | | | | | |  |  |
|  |  |  |  | Total Costs: | | | | | | | |  |  |
| 5029h | Which was the health care provider you visited? | Medical Doctor (including surgeon, gynaecologist, psychiatrist, ophthalmologist, etc) | | | | | | | | 1 | |  |  |
|  |  | Nurse/midwife | | | | | | | | 2 | |  |  |
|  |  | Dentist | | | | | | | | 3 | |  |  |
|  |  | Physiotherapist or chiropractor | | | | | | | | 4 | |  |  |
|  |  | Traditional medicine practitioner (*use local name*) | | | | | | | | 5 | |  |  |
|  |  | Pharmacist, druggist | | | | | | | | 6 | |  |  |
|  |  | Home health care worker | | | | | | | | 7 | |  |  |
|  |  | *DON'T KNOW* | | | | | | | | 8 | |  |  |

| Overall, what is the total expenditure on health care during the last 12 months? (this includes expenditure for all the members in the household for all episodes)  In patient care:  Outpatient care:  Are all your household members covered under any medical insurance: Yes No |
| --- |

| **SECTION 6: LIFE COURSE PERSPECTIVE** | | | | | | | | |
| --- | --- | --- | --- | --- | --- | --- | --- | --- |
| 6001 | How many brothers do you have? | | Ever born | | | 1 | |  |
|  |  |  | Surviving | | | 2 | |  |
| 6002 | How many sisters do you have? | | Ever born | | | 1 | |  |
|  |  |  | Surviving | | | 2 | |  |
| 6003 | Educational level of father when you were born (code) | | Non formal schooling | | | 1 | |  |
|  |  |  | Less than primary school | | | 2 | |  |
|  |  |  | Primary school completed | | | 3 | |  |
|  |  |  | Jnr. Secondary school completed | | | 4 | |  |
|  |  |  | Snr. Secondary school completed | | | 5 | |  |
|  |  |  | High school completed | | | 6 | |  |
|  |  |  | Tertiary school completed | | | 7 | |  |
|  |  |  | College/University completed | | | 8 | |  |
|  |  |  | Postgraduate degree | | | 9 | |  |
|  |  |  | Refused | | | 88 | |  |
| 6004 | Is your father alive? | | Yes | 1(If alive age?)  ______________ | | | | ***If no, go to Q6006*** |
|  |  |  | No | 2 | | | |  |
| 6005 | If not, your age at the time of your father’s death | | ___________________ | | | | |  |
|  | Cause of death In verbal terms | | Code= |  | | | |  |
| 6006 | Do you remember any major ailment that your father had during his life time? | | Yes | 1 | | | | ***If no, go to Q6008*** |
|  |  |  | No | 2 | | | |  |
| 6007 | If yes, name of ailment (CODE) | |  |  | | | |  |
| 6008 | Educational level of Mother when you were born | | Non formal schooling | | 1 | | |  |
|  |  |  | Less than primary school | | 2 | | |  |
|  |  |  | Primary school completed | | 3 | | |  |
|  |  |  | Jnr. Secondary school completed | | 4 | | |  |
|  |  |  | Snr. Secondary school completed | | 5 | | |  |
|  |  |  | High school completed | | 6 | | |  |
|  |  |  | Tertiary school completed | | 7 | | |  |
|  |  |  | College/University completed | | 8 | | |  |
|  |  |  | Postgraduate degree | | 9 | | |  |
|  |  |  | Refused | | 88 | | |  |
| 6009 | Is your mother alive? | | Yes | 1 (If alive age?)  _____________ | | | | ***If no, go to Q6012*** |
|  |  |  | No | 2 | | | |  |
| 6010 | If not, your age at the time of the death of your mother | | ___________________ | | | | |  |
| 6011 | Cause of death; In verbal Terms | | _________code |  | | | |  |
| 6012 | Do you remember any major ailment that your mother had during her life time? (Yes 01,No 02) | | Yes | 1 | | | | ***If no, go to Q6014*** |
|  |  |  | No | 2 | | | |  |
| 6013 | If ‘yes’ Name of ailment | |  | | | | |  |
| 6014 State activity status and occupation of your Father during your childhood?   \| 1. Government employee \| 1 \|  \| \| --- \| --- \| --- \| \| 1. Non-government employee \| 2 \| \| 1. Self-employed \| 3 \| \| 1. Non-paid/unpaid family helper \| 4 \| \| 1. Student \| 5 \| \| 1. Homemaker/house work \| 6 \| \| 1. Retired \| 7 \| \| 1. Unemployed (able to work) \| 8 \| \| 1. Unemployed (unable to work \| 9 \| \| 1. Other (Specify) \|  \| \| 1. Refused \| 88 \|   6015. State activity status and occupation of your Mother during your childhood?   \| 1. Government employee \| 1 \|  \| \| --- \| --- \| --- \| \| 1. Non-government employee \| 2 \| \| 1. Self-employed \| 3 \| \| 1. Non-paid/unpaid family helper \| 4 \| \| 1. Student \| 5 \| \| 1. Homemaker/house work \| 6 \| \| 1. Retired \| 7 \| \| 1. Unemployed (able to work) \| 8 \| \| 1. Unemployed (unable to work \| 9 \| \| 1. Other (Specify) \|  \| \| 1. Refused \| 88 \| | | | | | | | | |
| **ADDITIONAL LIFE COURSE QUESTIONS**  Can you re-collect more about your past based broadly during your childhood? | | | | | | | | |
| 6016 | Was any member of the household literate (Yes 01, No 02) | | | | | |  | |
| 6017 | Was your village connected with the outside by road (Yes-01; No-02) | | | | | |  | |
| 6018 | If connected by bus, distance from your residence to the nearest bus stop (in kms.) | | | | | |  | |
| 6019 | Distance from your residence to the nearest school (in km.) | | | | | |  | |
| 6020 | Distance from your residence to the nearest market (in km.) | | | | | |  | |
| 6021 | Distance from your residence to the nearest hospital/dispensary (in km.) | | | | | |  | |
| 6022 | Distance from your residence to the nearest urban/village centre (in km.) | | | | | |  | |
| 6023 | Have your life been stressful? (Yes-01, No-02) | | | | | |  | |
| 6024 | With whom were you living for most of the time? (father only-01, mother only-02, father and mother together-03, with own family-04, with grandfather/grandmother-05, with siblings- 06, alone-07, others-08 (specify) | | | | | |  | |
| 6025 | Kind of food taken (vegetarian-01, non-vegetarian-02) | | | | | |  | |
| 6026a | If you are a non-vegetarian, did you use (regularly 01, mostly 02, occasionally 03, sparingly 04, never 05) | 1. Fish | | | | |  | |
|  |  | 1. Beef | | | | |  |  |
|  |  | (c) mutton | | | | |  |  |
|  |  | (d) chicken | | | | |  |  |
| 6076b | Did you have at least one square meal per day throughout the year? (01 yes, 02 no) | | | | | |  | |
| 6076c | If yes, how many | | | | | |  | |
| 6077 | Do you feel that your food intake was adequate for your needs? (more than adequate-01, adequate-02, inadequate-03) | | | | | |  | |
| 6078 | How did you feel of your health (below average-01, average-02, above average 03) | | | | | |  | |
| 6079 | Have you ever participated in games/sports/ Done Physical Exercise/ Participated or any manual work? (Regularly-01, Mostly-02, Occassionally-03, rarely-04, Never-05) | | | | | |  | |
| 6080 | Do you remember any major ailment you suffered? (Yes-01, No-02) | | | | | |  | |
| 6081 | If yes, report the major ailment? | Name | | | | |  | |
|  |  | Code | | | | |  |  |

| **SECTION 7: ANTHROPOMETRIC MEASUREMENTS** | | | |
| --- | --- | --- | --- |
| **Anthropometrics: Performance Tests and Biomarker** | | | |
| **Anthropometric Measurements**  I would now like to measure how tall you are. To measure your height I need you to please take off your shoes. Put your feet and heels close together, stand straight and look forward standing with your back, head and heels touching the wall. Look straight ahead. | | | |
| 7001 | Measured height in centimeters: | Centimeters:  997 Refused  998 Not able |  |
| Now we want to measure your weight - could you please keep your shoes off and step on this scale. We will also measure your waist and hips using a tape measure. | | | |
| 7002 | Measured weight in centimetres | Kilograms:  997 Refused  998 Not able |  |
| 7003 | Waist circumference in centimeters:  *INTERVIEWER: Identify the top of the hip bone and make sure the tape measure is parallel to the floor all the way around the body* | Centimeters:  997 Refused  998 Not able |  |
| 7004 | Hip circumference in centimeters:  *INTERVIEWER: Measure at the maximum around the body circumference of the hips and make sure the tape measure is parallel to the floor all the way* | Centimeters:  997 Refused  998 Not Able |  |
| Now you can put your shoes back on, if you wish | | | |
| Notes: | | | |

**END OF INTERVIEW: Hours……….. Minutes…………**
