# Supplementary material for: Socioeconomic inequalities in non-communicable disease risk factors in Botswana: a cross-sectional study
Source: BMC Public Health. 2019 Aug 7;19:1060. doi: 10.1186/s12889-019-7405-x (PMC6686547; doi:10.1186/s12889-019-7405-x)
Supplement: Supplementary file 2 — Questionnaires (DOCX 68 kb) [file 12889_2019_7405_MOESM2_ESM.docx]

**Additional file 2**

**Methodology for the NCDs study,2016 (Chapter 3)**

**Data sources**

The study on ‘Chronic Non communicable Diseases in Botswana; A study on chronic disease prevalence, Health care Utilization, Health Expenditure and the Life course used a multistage probability sampling technique. The study was conducted in March 2016 and collected additional information on CNCDs in Botswana beyond what is provided for by the WHO NCDs surveillance survey. The NCD study collected information on other chronic diseases and their risk factors in the country. It also collected information on health care utilization, health expenditure and indicators about the childhood experiences associated with NCDs. Self-reported data on several NCDs as classified by the WHO classification of diseases and their associated risk factors was collected. The collection of self-reported morbidity on diabetes and hypertension would serve as baseline for comparison between clinical and self-reported prevalence of CNCDs in Botswana.

**Design and Selection of the Sample**

The study on ‘Chronic Non communicable Diseases in Botswana; A study on chronic disease prevalence, Health care Utilization, Health Expenditure and the Life course’’ adopted a representative cross-sectional descriptive study design as well as a qualitative design. The survey was carried in selected urban and rural areas of Botswana, among males and females aged 15 years and over[[1]](#footnote-1). A list of districts, localities and enumeration areas (EAs) together with their households was made based on the 2011 Botswana Population and Housing sampling frame. The survey used a multi-stage probability sampling technique, where first the population was stratified into cities and towns, urban villages and rural settlements. A listing of all 26 census districts in each strata was made at the initial stage, and from these districts a total of all 4845 EAs were listed for rural and urban localities. At the second stage, localities in urban and rural districts were randomly selected. A third and fourth stage comprised a random selection of EAs and Households in that order. Lastly, individuals aged 15 years and over were selected for interview from the list of households with persons 15 years and over.

**Sampling Procedure**

Using the multistage probability sampling, census districts for Botswana were divided into rural and urban clusters at the first step. Urban districts were further divided into cities, towns and urban villages; while rural clusters were randomly selected (thus rural settlements in lands area, cattle posts, freehold farms, mixture of lands and cattle posts, and camp or other locality where type of locality is not .stated were excluded from the sample).

From cities and towns, the following were randomly selected: Gaborone, Jwaneng and Selebi Phikwe. For the urban village’s strata the villages of: Kanye, Letlhakeng, Maitengwe, Maun, Mochudi, Molepolole, Serowe, Tsabong, Tlokweng, Tutume, Kopong, Mmadinare, Lerala, Gumare, and Thamaga were randomly selected. While the rural villages strata included the following randomly selected villages; Nthanthe, Ditshegwane, Senete, Sehithwa, Mathubudukwane, Serinane, Moiyabana, Omaweneno, Dikwididi, Makuta, Kgope, Tobane, Maunatlala, Etsha 6 and Kubung.

Enumeration areas were selected using probability proportional to size sampling method for the different strata and localities. The number of enumeration areas to be selected in each stratum will be fixed according to the total sample size distributed to the district as per the procedure explained below.

***Sample Size calculation***

The total sample size has been determined using the formula given below and then allocated it across the strata. Generally in most of the household surveys, the strategy is usually considered. The size of the sample is one of the important parameter of the sample design, because it affects the *precision*, the *cost* and *duration* of the survey more than any other factor. The following factors are considered in calculation of sample size. These are:

1. Margin of error, (5%)
2. Level of confidence (95%)
3. Design effect[[2]](#footnote-2) (1.75)
4. Expected response rate (80%)

For the large N, ignoring the adjustment for the size of the population [N/(N + n)]; the required sample size is

$n_{1}=\frac{D Z_{\alpha}^{2} p q}{e^{2} r} =\frac{1.75 x 1.96^{2} x 0.5 x0.5}{0.05^{2} x 0.80} =$ 840

Where,

- n_1_ = required sample size for each place of residence category.
- D= Design effect
- $Z_{\alpha}$= value of Z which provides α/2 in each tail of normal curve, if α is .05 then the 2-tailed Z is 1.96
- p= proportion of indicator in the population; q= (1-p). It is taken as 0.5 as it gives the maximum variability in the population.
- e= margin of error
- r= expected response rate

The minimum sample size required is 840 households. We arrived at the final sample size of 1280, increasing the minimum required sample size by 50% to account for better representation in the three strata such as cities and towns, urban villages and rural areas and to produce estimates for Botswana and for broad socioeconomic categories. The final selection of respondents for inclusion in the study was that 28.5% of the respondents were recruited from cities and towns, 47.3% from urban villages and 24.1% from rural villages. This is proportionate to the size of the population as estimated during the 2011 Botswana Population and Housing Census.

Hence the total respondents from towns and cities=28.5/100*1280=365 respondents; 47.3/100*1280=606 respondents from urban villages, and a total of 24.1/100*1280=309 respondents from rural villages were targeted.

Furthermore, the selection of the sample within each enumeration area is calculated in relation to the proportion of population in that district. E.g. for Gaborone = 178654/226649*365=288 (see table1).

Table 4: Summary of sampled districts with their enumeration areas and proportion of study respondents.

| **Proposed Localities** | **Population aged 15-65 years (2011 Census)** | **Proposed Number of EA's** | **Proposed Number of Population Aged 15-65 years** |
| --- | --- | --- | --- |
| **Cities and Towns (324291)** | **226049** | **18** | **365** |
| Gaborone | 178654 | 14 | 288 |
| Selebi-Phikwe | 34093 | 3 | 55 |
| Jwaneng | 13302 | 1 | 21 |
|  |  |  |  |
| **Urban Villages (538585)** | **271900** | **30** | **606** |
| Kanye | 31108 | 3 | 69 |
| Letlhakeng | 5439 | 1 | 12 |
| Maitengwe | 2969 | 0 | 7 |
| Maun | 40796 | 5 | 91 |
| Mochudi | 29331 | 3 | 65 |
| Molepolole | 43103 | 5 | 96 |
| Serowe | 35614 | 4 | 79 |
| Tsabong | 6131 | 1 | 14 |
| Tlokweng | 28211 | 3 | 63 |
| Tutume | 10626 | 1 | 24 |
| Kopong | 6856 | 1 | 15 |
| Mmadimare | 9192 | 1 | 20 |
| Lerala | 3943 | 0 | 9 |
| Gumare | 5180 | 1 | 12 |
| Thamaga | 13401 | 1 | 30 |
|  |  |  |  |
| **Rural Villages (274363)** | **21702** | **15** | **309** |
| Nthanthe | 1321 | 1 | 19 |
| Ditshegwane | 1209 | 1 | 17 |
| Senete | 1415 | 1 | 20 |
| Sehithwa | 2928 | 2 | 42 |
| Mathubudukwane | 1233 | 1 | 18 |
| Serinane | 358 | 0 | 5 |
| Moiyabana | 3014 | 2 | 43 |
| Werda | 1905 | 1 | 27 |
| Dikwididi | 225 | 0 | 3 |
| Makuta | 464 | 0 | 7 |
| Kgope | 521 | 0 | 7 |
| Tobane | 1455 | 1 | 21 |
| Maunatlala | 2871 | 2 | 41 |
| Etsha 6 | 2783 | 2 | 40 |
| Kubung | 188 | 0 | 3 |

For each selected EA, 20 households were selected using systematic sampling method. This followed guidelines used in most Demographic Health Surveys where 20-25 households (hhs) were selected from the primary sampling units (PSUs). For instance, in the case of cities and town; 365/20=18 EAs. The above procedure was followed for all districts, where each of the sampled EAs, 20 households were selected using systematic sampling method. The Kish grid was used to select the eligible respondents from the selected households. Thus, once a household is selected, the interviewer created a listing (sampling frame) of all the persons in the household that are eligible for the interview process. This listing includes the name of the person, their gender, their relationship to the head of the household and their age. Once the listing was done, each eligible member was assigned a unique number. Then using a randomized response table a particular member was chosen for the interview.

**Survey Instruments**

A population based survey comprising of quantitative and qualitative[[3]](#footnote-3) data collection approaches was proposed.

1. *Design of NCD study Instruments*

The adapted instruments in the NCD study were based on several resources. These were mainly from the WHO Study on Global Ageing and Adult Health (SAGE), and WHO STEPS Survey. These were then reviewed and subsequently adopted by the research team. The review took into account the recommendations by the World Health Organization on undertaking population-based surveys.

1. *Quantitative data collection instrument*

This employed a structured questionnaire and collected data from a representative sample of Batswana using the interview method. The focus of the interview was on the following key issues:

1. Socioeconomic and demographic information.
2. Housing characteristics.
3. Known or perceived risk factors associated with self or an individual known to have a NCD condition.
4. Health care utilization and health expenditure patterns associated with CNCDs.
5. Level of awareness and perceptions of the population regarding the listed non communicable diseases.
6. Childhood socioeconomic status and adolescence behavior on prevalence of CNCDs and their risk factors in later life.
7. Anthropometric Measurements.
8. *Qualitative data collection instrument -* *Key informant interviews*

This method sought to collect data on perceptions regarding the readiness or preparedness of the Botswana health system in dealing with non-communicable diseases. This method was used to collect data from significant actors in the community and health facilities who are expected to be knowledgeable on the disease load and profiles of in their communities. Health facilities managers were contacted for information regarding reported NCDs to their facilities and the facilities readiness to address reported NCD conditions.

**Data Collection Procedures and Management**

Scientific procedures for data collection and management are important for data quality and therefore its utilization. This is particularly relevant where scientific research undertaking has an expectation to inform policy direction. The quality and utility of a CNCD study data was dependent on the manuals, control forms and questionnaires used in the survey. The CNCD study as a consequence opted to use validated instruments and manuals that were informed by past research and United Nations bodies such as WHO.

**Data collection Instruments**

In the CNCD study two instruments were administered (a questionnaire and interview guide). Their design was made to serve the purpose of: (i) of extracting specific [information](http://www.businessdictionary.com/definition/information.html), (ii) collecting the appropriate [data](http://www.businessdictionary.com/definition/data.html), (iii) making data comparable and amenable to [analysis](http://www.businessdictionary.com/definition/analysis.html), (iv) minimizing [bias](http://www.businessdictionary.com/definition/bias.html) in formulating and asking question, and (V) making questions engaging and varied. Careful consideration was accorded the type of instrument regarding; its format, wording and sequencing of the questions, the method of enumeration, the data being collected, and how the data will be processed.

**Preparatory Activities**

A number of preparatory activities were undertaken before the commencement of the study on CNCD. These were: (i) communication, publicity and advocacy and (ii) recruitment, training and deployment of the field research staff.

1. ***Communication, Publicity and Advocacy***

Prior to the field work survey the research team made contact with District Administration/Office, Local Authority and Tribal Administration to make them understand and appreciate the object of the study, importance of the study and use of the data and underscoring the confidentiality of responses, and when enumeration is scheduled to take place in their of area of jurisdiction.

On arrival at the survey sites, the first point of entry was to make contact with the tribal authority to make them aware of the presence of enumerators in their village/locale as well as to make an appointment for in-depth interviews with them or local structures (e.g. VDC, VHC etc).

1. ***Field staff recruitment, training and deployment***

In endeavour to adhere to the aforesaid principles, the study recruited ten (10) research assistants with a minimum qualification of a bachelor’s degree (in the social sciences), two (2) field supervisors and one (1) project administrator. They were then trained on field survey methodology, interview skills and research ethics. These were based on the WHO Training Manual on the Study on Global Ageing and Adult Health (SAGE and other documents were used to reinforce this.

Two (2) teams of 6 people each were deployed with enough material to cover their target areas of enumeration. The supervisors in these teams were tasked with receiving, holding, dispatching, collecting and returning to the Project Administrator all the project documents and materials

**Testing of CNCD Instruments**

The pretesting of the instrument prior to the nationwide field survey is common practice. The CNCD study undertook this exercise in one of the sampled settlements (Dikwididi, Kgatleng District) to familiarize the research assistant’s with the utility of the questionnaire focusing on: the suitability of intended survey questions, their formulation and the instructions provided, as well as the suitability of the questionnaire design. Testing of the instruments revealed information relating to the average duration of administering questionnaires (approximately 60 minutes), which questions respondents found relevant/irrelevant/intrusive/less so, and how many questionnaires were successfully completed in a specified time (8/13=62 percent). Furthermore, it gave us insights into how the field work can be organized, what further training was needed to improve the skills of the interviewers as well as the extent of respondent burden.

**Enumeration Strategy**

The enumeration strategy for the CNCD study encompassed delineation of activities and definition of responsibilities, type and method of enumeration so as to better execute field operations.

1. *Activities and Responsibilitie*s

In achieving the overall goal of the study, the following principles were encouraged:

1. Full coverage of sampled localities and EAs;
2. Confidentiality;
3. Communication and advocacy
4. Accountability and
5. Consistency of procedures in all the survey localities.
6. *Type of enumeration*

The CNCD study adopted modified de facto type of enumeration whereby respondents 15-65 years old were enumerated at the place where they were found at the time of survey. This, however, excluded members of the household who were not usual residents.

1. *Method of enumeration*

The method of enumeration adopted by CNCD study was the interviewer (canvasser) method.

The CNCD study adopted this approach given the budget, content and scope of the study. In enumerating an EA, first a coin was tossed to decide the cardinal point (see Figure 1 for illustration) where the enumeration will start. The first household to be interviewed was determined using the day code. For example, on the 25^th^ March 2016 – the first household to be enumerated would be the 7^th^ household from the farthest point of the EA. This code was arrived at by adding the digits 2 and 5. For subsequent selection of a household, a sampling interval was determined based on the size of the EA and the total number of interviews per EA.

**Figure 3.1: Cardinal Directions**


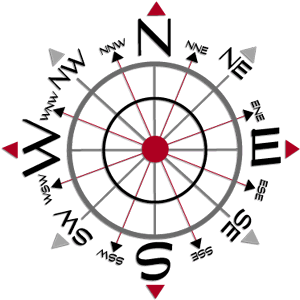


***Problems that came up during the Survey***

On the basis of the observations made on the pilot and main survey, though some respondents found some questions of no relevance and intrusive, overall there were no major weaknesses in the questionnaire or in the enumeration procedure that might affect the quality of data. However, the data analyst should be cautious in their interpretation of data and be on the lookout for emerging patterns on household assets and income. This is because observations by field research assistants were that some respondents withheld information with the belief that if they were to disclose their assets and/or income, they may not get financial assistance from government.

***Quality assurance***

To ensure that data collected is of high quality, a number of quality assurance mechanisms were adopted:

1. Recruitment and training of experienced research assistants
2. Pre-test of data collection tools
3. Field and office editing of completed tools by data collection supervisors
4. Data cleaning: This was done before analysis to ensure consistency and completeness.

***Implications of the proposed study***

The proposed study is vital for both policy and intellectual reasons. CNCDs feature in many health policy agendas worldwide and in the post 2015 health agenda. For intellectual reasons because there is need for prospects of developing new methods of CNCDs research in Botswana based on her unique health context. The proposed study will help to inform and target effective interventions for CNCDs and increase awareness about CNCDs.

**Response Rate**

Summary of Sampled Districts with their Enumeration Areas, Proposed Population to be Interviewed, Number Interviewed and Response Rate

| **Proposed Localities** | **Population aged 15-65 years (2011 Census)** | **Proposed Number of EA's** | **Proposed Number of Population Aged 15-65 years** | **Number of Interviews Successfully Conducted** | **Response rate** |
| --- | --- | --- | --- | --- | --- |
| **Cities and Towns (324291)** | **226049** | **18** | **365** | **355** | **97.3** |
| Gaborone | 178654 | 14 | 288 | 276 | 95.8 |
| Selebi Phikwe | 34093 | 3 | 55 | 55 | 100.0 |
| Jwaneng | 13302 | 1 | 21 | 24 | 114.3 |
|  |  |  |  |  |  |
| **Urban Villages (538585)** | **271900** | **30** | **606** | **534** | **88.1** |
| Kanye | 31108 | 3 | 69 | 74 | 107.2 |
| Letlhakeng | 5439 | 1 | 12 | 12 | 100.0 |
| Maitengwe | 2969 | 0 | 7 | 9 | 128.6 |
| Maun | 40796 | 5 | 91 | 68 | 74.7 |
| Mochudi | 29331 | 3 | 65 | 65 | 100.0 |
| Molepolole | 43103 | 5 | 96 | 54 | 56.3 |
| Serowe | 35614 | 4 | 79 | 80 | 101.3 |
| Tsabong | 6131 | 1 | 14 | 14 | 100.0 |
| Tlokweng | 28211 | 3 | 63 | 50 | 79.4 |
| Tutume | 10626 | 1 | 24 | 20 | 83.3 |
| Kopong | 6856 | 1 | 15 | 15 | 100.0 |
| Mmadinare | 9192 | 1 | 20 | 20 | 100.0 |
| Lerala | 3943 | 0 | 9 | 10 | 111.1 |
| Gumare | 5180 | 1 | 12 | 19 | 158.3 |
| Thamaga | 13401 | 1 | 30 | 24 | 80.0 |
|  |  |  |  |  |  |
| **Rural Villages (274363)** | **21702** | **15** | **309** | **288** | **93.2** |
| Nthanthe | 1321 | 1 | 19 | 17 | 89.5 |
| Ditshegwane | 1209 | 1 | 17 | 13 | 76.5 |
| Senete | 1415 | 1 | 20 | 20 | 100.0 |
| Sehithwa | 2928 | 2 | 42 | 34 | 81.0 |
| Mathubudukwane | 1233 | 1 | 18 | 20 | 111.1 |
| Serinane | 358 | 0 | 5 | 6 | 120.0 |
| Moiyabana | 3014 | 2 | 43 | 41 | 95.3 |
| Werda | 1905 | 1 | 27 | 18 | 66.7 |
| Dikwididi | 225 | 0 | 3 | 12 | 400.0 |
| Makuta | 464 | 0 | 7 | 5 | 71.4 |
| Kgope | 521 | 0 | 7 | 7 | 100.0 |
| Tobane | 1455 | 1 | 21 | 20 | 95.2 |
| Maunatlala | 2871 | 2 | 41 | 40 | 97.6 |
| Etsha 6 | 2783 | 2 | 40 | 30 | 75.0 |
| Kubung | 188 | 0 | 3 | 5 | 166.7 |
| National (1137239) | 519651 | 63 | 1280 | 1178 | 92.0 |

**Ethical Clearance**

All ethical clearance formalities were completed before the start of the study. The study proposal along with the necessary documents were submitted to Institutional Review Board of the University of Botswana for ethical clearance. Privacy and confidentiality of highest standard shall was maintained by treating all respondents as anonymous, and none of the respondents names were mentioned or implied when presenting findings of the study.

**Internal Validity**

The CNCD has adopted a cohort research design to:

1. Assess the magnitude and patterns of the listed CNCDs[[4]](#footnote-4) in Botswana;
2. Assess the levels and patterns of behavioral risk factors for CNCDs.
3. Investigate the health care utilization associated with CNCDs.
4. Investigate the health expenditure associated with CNCDs and its implications on household poverty.
5. Investigate the level of awareness and perceptions of the population regarding the listed non communicable diseases;

These types of survey designs are poor in internal validity because of their inability to manipulate the independent variable (cause), and because cause and effect are measured at the same point in time which defeats temporal precedence making it equally likely that the expected effect might have influenced the expected cause rather than the reverse ([Bhattacherjee, 2012](#_ENREF_1)). Despite this, the use of a cohort design allows for measurement of potential causes before the outcome has occurred, they can demonstrate that these “causes” preceded the outcome, thereby avoiding the debate as to which is the cause and which is the effect (["Organizing Your Social Sciences Research Paper: Types of Research Designs,"](#_ENREF_2)).

1. Qualitative data will be collected to assess the readiness or preparedness of the Botswana health system in dealing with non-communicable diseases. [↑](#footnote-ref-1)
2. The design effect is the ratio of the sampling variance of an estimator under a given design to the sampling variance of an estimator under SRS of the same sample size. Therefore, for a simple random sample design, deff = 1, and usually deff ≤ 1 for a stratified sample design and deff ≥ 1 for a cluster sample design. [↑](#footnote-ref-2)
3. [↑](#footnote-ref-3)
4. WHO Classification of diseases was used for this purpose. [↑](#footnote-ref-4)
