# Supplementary material for: Socioeconomic inequalities in non-communicable disease risk factors in Botswana: a cross-sectional study
Source: BMC Public Health. 2019 Aug 7;19:1060. doi: 10.1186/s12889-019-7405-x (PMC6686547; doi:10.1186/s12889-019-7405-x)
Supplement: Supplementary file 3 — Decomposition analysis equation (DOCX 23 kb) [file 12889_2019_7405_MOESM3_ESM.docx]

Additional file 3_ Decomposition analysis equation

The concentration index and curves for NCD risk factors were derived from the following equation.

For a discrete living standards variable, the index is defined as;

$$1. ∁=\frac{2}{n\mu h}\sum_{i-1}^{n} h_{i}R_{i}-1-\frac{1}{n}$$

Where: h*_i_* is the risk factor, μ*_h_* is its mean, and R*_i_*=*i*/n is the fractional rank of individual *i* in the wealth status distribution, with *i*= 1 for the poorest and *i*= n for the richest^29^. The concentration index depends on the relationship between NCD risk factors and the rank of the wealth status variable (h*_i_*r*_i_*) ^28^. The value judgments implicit in the index are seen when the index is written as:

$$2. ∁=1-\frac{2}{n\mu}\sum_{i-1}^{n} h_{i}(1-R_{i})$$

The quantity h*i*/n*_μ_* is the ith person’s share of a risk factor. This is then weighted in the summation by twice the complement of the person’s fractional rank, that is, 2 (1–R*i*). So the poorest person has the share of a risk factor weighted by a number close to two. The weights decline in a stepwise fashion, reaching a number close to 0 for the richest person. The extended concentration index is then 1 minus the sum of these weighted health shares.

$$3. C\left( v \right)=1-\frac{v}{n\mu}\sum_{i-1}^{n} {h_{i}(1-R_{i})}^{(v-1)}$$

Where: *v* is the inequality-aversion parameter (the weight attached to the *i*th person’s risk factor share), h*i*/nμ, is now equal to v (1–R*i*)(*v−1*), rather than by 2 (1–R*i*).When *v*= 1 everyone’s risk factor is weighted equally. As *v* is raised above 1, the weight attached to a risk factor for a very poor person rises, and the weight attached to a risk factor for a person above the 55^th^ percentile decreases. Achievement Index (AI) was used to reflect average level of a specified risk factor. The index is therefore defined as a weighted average of a specified risk factor in the sample with higher weights attached to the poor than to better off. The index is given as:

4. $1\left( v \right)=\frac{1}{n}\sum_{i-1}^{n} h_{i}v{(1-R_{i})}^{(v-1)}$

This index can be shown to be equal to:

$$1\left( v \right)=\mu\begin{matrix} [1-C\left( v \right)] \end{matrix}$$

When h is a measure of good health, high values of I *(v)* are considered good and C *(v)*> 0 (good health is higher among the non-poor). If a specified risk factor declines monotonically with wealth status, the greater is the degree of inequality aversion, and the greater is the wedge between the mean (*μ*) and the value of the index I (*v*) ^28^. Indirect method of standardization was used to reflect differences across socioeconomic groups while controlling other determinants of a specified risk factor variable. The standardizing variables are those correlated with the wealth status measure and that of the risk factors from existing empirical literature. Such standardization provides a way to remove components of inequalities from socioeconomic related inequalities and describe the distribution of the NCD risk factors by socioeconomic status conditional on other demographic, socio-economic factors^28.^

$$5. y=\propto+\sum_{j} \beta_{j}X_{i}j+\sum_{k} ykZkj+\varepsilon_{i}$$

Where:$y$*i* is the risk factor variable for the *ith* individual; and α, β and $y$are parameter vectors, xj are confounding variables used to standardize, and z_k_ are non-confounding variables for which we do not want to standardize but do want to control for in order to estimate partial correlations with the confounding variables.*α*, *β_j_* and *y_k_* parameter estimates of individual values of the confounding variables (x*ji*), and sample means of the non-confounding variables (zk) are then used to obtain the predicted values of the health indicator *γi*.

Estimates of indirectly standardized health outcomes are then computed by the difference between actual and predicted outcomes plus the overall sample mean^29^. Socioeconomic related inequalities were decomposed into the contributions of individual factors to wealth- related NCD risk factors inequality, in which each contribution is the product of the sensitivity of risk factor with respect to that factor and the degree of socioeconomic-related inequality in that factor.

6. $Y_{i}=\propto\sum k\beta_{k}Xk_{i}+\epsilon_{i}$

Where: Yi= 1 for the specified risk factor variable, Xk a set of exogenous determinants of that risk factor variable andβk coefficient determinant Xk, and €I is random error term.
